# Mitochondrial alternative oxidase‐dependent autophagy involved in ethylene‐mediated drought tolerance in *Solanum lycopersicum*

**DOI:** 10.1111/pbi.12939

**Published:** 2018-05-24

**Authors:** Tong Zhu, Lijuan Zou, Yan Li, Xiuhong Yao, Fei Xu, Xingguang Deng, Dawei Zhang, Honghui Lin

**Affiliations:** ^1^ Key Laboratory of Bio‐Resources and Eco‐Environment of Ministry of Education College of Life Sciences Sichuan University Chengdu Sichuan China; ^2^ Life Science and Technology College and Ecological Security and Protection Key Laboratory of Sichuan Province Mianyang Normal University Mianyang China; ^3^ Life Science and Biotechnology Wuhan Bioengineering Institute Wuhan China

**Keywords:** ethylene, alternative oxidase, autophagy, drought, reactive oxygen species, *Solanum lycopersicum*

## Abstract

Mitochondrial alternative oxidase (AOX) is involved in a large number of plant physiological processes, such as growth, development and stress responses; however, the exact role of AOX in response to drought remains unclear. In our study, we provide solid evidences that the activated AOX capacity positively involved in ethylene‐induced drought tolerance, in tomato (*Solanum lycopersicum*), accompanied by the changing level of hydrogen peroxide (H_2_O_2_) and autophagy. In *AOX1a*‐RNAi plants, the ethylene‐induced drought tolerance was aggravated and associated with decreasing level of autophagy. The H_2_O_2_ level was relatively higher in *AOX1a*‐RNAi plants, whereas it was lower in *AOX1a*‐overexpressing (35S‐*AOX1a‐OE*) plants after 1‐(aminocarbonyl)‐1‐cyclopropanecarboxylic acid (ACC) pretreatment in the 14th day under drought stress. Interestingly, the accumulation of autophagosome was accompanied by the changing level of reactive oxygen species (ROS) in AOX transgenic tomato under drought stress whether or not pretreated with ACC. Pharmacological scavenging of H_2_O_2_ accumulation in *AOX1a*‐RNAi (*aox19*) stimulated autophagy acceleration under drought stress, and it seems that AOX‐dependent ROS signalling is critical in triggering autophagy. Lower levels of ROS signalling positively induce autophagy activity, whereas higher ROS level would lead to rapid programmed cell death (PCD), especially in ethylene‐mediated drought tolerance. Moreover, ethylene‐induced autophagy during drought stress also can be through ERF5 binding to the promoters of *ATG8d* and *ATG18h*. These results demonstrated that AOX plays an essential role in ethylene‐induced drought tolerance and also played important roles in mediating autophagy generation via balancing ROS level.

## Introduction

Nowadays, the increasing demand for food and vegetables was still one of the big challenges for global population growth and economic development, especially in developing countries. Lack of water led to reduced crop yields and economic losses in drought regions. Plant drought tolerance is a meaningful measure to reduce the impact of drought on crop productivity. In order to survive in adversities, plants have evolved sophisticated mechanisms to degrade and recycle useless or valuable intracellular components when they perceive the signal of environmental deterioration. Autophagy (also known as macroautophagy), an eukaryotic cell degrading and recycling process, plays an important role in the cellular response to abiotic stresses (Bassham, 2007; Liu *et al*., 2009; Lv *et al*., 2014; Slavikova *et al*., 2008). In brief, the double‐membrane vesicle which is called autophagosome could enclose any cytoplasmic components and deliver them to the vacuole/lysosome; after then, the toxic components and required molecules are gradually degraded and recycled in organelles (He and Klionsky, 2009; Liu and Bassham, 2012). Autophagy‐related genes (*ATG*) have been found in almost all eukaryotes, and they are quite conserved through evolution (Pérez‐Pérez *et al*., 2012). There are more than 30 autophagy‐related (*ATG*) genes in *Arabidopsis*. These *ATG* genes and their homologs participate in main autophagy process which have been identified in different kinds of plants over the last 20 years (Kim *et al*., 2012; Kwon and Park, 2008). Recent study reported that there were more than 25 *ATG* genes in tomato and several *ATG* families were proved to take part in the process of drought and salt resistance (Liu *et al*., 2009; Wang *et al*., 2015).

Increasing studies have demonstrated reactive oxygen species (ROS) and autophagy have been associated with cell death under environmental stresses (Henry *et al*., 2015; Pérez‐Pérez *et al*., 2012). There were also some reports showing a complex crosstalk between ROS and autophagy that is partly mediated by hormone signalling cascades in the regulation of flowering, plant senescence and stress tolerance (Love *et al*., 2008; Shibuya *et al*., 2013; Slavikova *et al*., 2008; Yoshimoto *et al*., 2009). For instance, jasmonic acid, ethylene and salicylic acid play important but antagonistic roles in rapid apoptosis‐like PCD (programmed cell death) (Love *et al*., 2008). Evidences show that ROS also regulate starvation‐induced autophagy, which is clearly a survival pathway (Scherz‐Shouval and Elazar, 2007). Recent findings show that mitochondrial generation of ROS is a trigger for autophagy (Chen and Gibson, 2008; Lee *et al*., 2012; Scherz‐Shouval and Elazar, 2007). The mitochondrial electron transport chain and the peroxisomes are primary sources of ROS production in most eukaryotes. Studies indicate that plant mitochondria are at a cross‐point in the signalling pathways involving ROS, especially those concerning cell death (Jones, 2000). ROS, specifically, H_2_O_2_, are reported to participate in inducing autophagy and autophagic cell death (Scherz‐Shouval and Elazar, 2007).

In order to withstand the redundant ROS generation, plant cells have evolved sophisticated defence mechanisms to avoid organelle damage. For example, mitochondrial redox status is maintained by dissipation of excessive electron flow via several systems such as AOX, plant uncoupling proteins (UCPs) and plant mitochondrial potassium channel (Pmito‐KATP). Studies have shown that AOX functions to balance the energy stability and keep the electron transport chain (ETC) flowing through mitochondrial by limiting the formation of mitochondrial ROS (Panda *et al*., 2013; Wang *et al*., 2012; Xu *et al*., 2012a). It is well known that the ETC in the mitochondria could be over‐reduced, accompanying with the generation of superoxide (O_2_^.−^), especially in stress condition (Deng *et al*., 2015; Minibayeva *et al*., 2012; Pu *et al*., 2015). Expression of AOX is activated not only by stress conditions but also by several phytohormone stimuli. Previous researches have reported that ethylene‐dependent pathways are required for O_3_‐induced up‐regulation of *AOX1a* (Ederli *et al*., 2006). Ethylene treatment could also enhance the AOX capacity under stress condition (Wang *et al*., 2010). Other phytohormones, like brassinosteroids, could enhance the alternative respiratory pathway in *Nicotiana benthamiana* in different abiotic stresses (Deng *et al*., 2015). In plant, AOX‐dependent defence mechanism seems to be continuous and indispensable, in particular in response to various stresses (Mittler, 2002; Xu *et al*., 2012a). Furthermore, evidences have shown that up‐regulated AOX pathway enhanced drought tolerance in wheat and the absence of *AOX1a* in Arabidopsis resulted in severe damage under drought stress (Bartoli *et al*., 2005; Giraud *et al*., 2008).

Studies have also shown that ethylene signal could also mediate induction of *GmATG8i* in soybean plants under starvation stress (Du *et al*., 2014; Okuda *et al*., 2011). Although ABA‐dependent pathway is important in plant drought response, an ABA‐independent pathway probably played a role in autophagy induction during drought stress (Liu *et al*., 2009). Until now, the roles of mitochondrial AOX and autophagy in the drought response have been investigated independently, and it was observed that both regulatory systems share some common regulatory and response genes. So we speculate the AOX‐dependent ROS signalling and autophagy pathway might have as critical roles in ethylene‐mediated tomato drought responses. Furthermore, the possible relationship between AOX and autophagy in alleviating ethylene‐induced drought tolerance was investigated.

## Results

### Ethylene‐induced AOX capacity and drought responses

**Figure 1:**
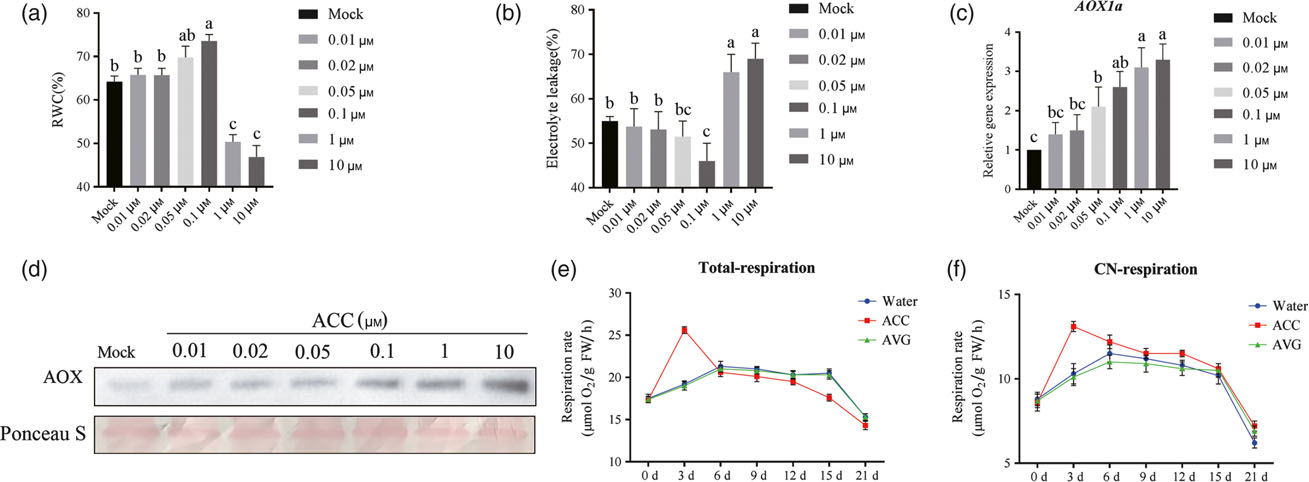
Ethylene‐induced AOX capacity and drought tolerance. (a, b) Relative water content (RWC) (a) and electrolyte leakage (EL) (b) of terminal leaflets in different concentration of ACC‐pretreated tomato plants measured in the 14th day under drought stress. (c, d) Induction of *SlAOX1a* expression (c) and AOX protein levels (d) of the terminal leaflets were determined immediately in the 14th day of drought after mock or ACC pretreatment. Total RNA and protein were isolated from leaf samples at the same times. Ponceau S‐stained membranes are shown below the blots to indicate the amount of protein loaded per lane. (e, f) Total respiration (e) and alternative respiration (f) were measured in the indicated time points under drought condition. All data are presented as the means of at least three biological replicates (±SE). Means with the same letter did not significantly differ at *P* < 0.05 according to Duncan multiple range tests. Three independent experiments were performed with similar results.

To assess the effect of ethylene in tomato drought response, tomato seedlings were pretreated with water, various concentrations of 1‐aminocyclopropane‐1‐carboxylic acid (ACC, the precursor of ethylene) and then withholding water for 2 weeks. After 14 days, the change in relative water content (Figure 1a) and electrolyte leakage (Figure 1b) were examined. The results showed that relative higher concentration (e.g. >1 μm) of ACC‐pretreated tomato exhibited more significant drought sensitivity compared with control whereas 0.1 μm ACC showed an enhanced drought tolerance in the 14th day after withholding water. We found that ACC pretreatment caused an induction in either *AOX1a* transcript levels or AOX protein levels under drought (Figure 1c and d). Concentrations of ACC from 0.01 to 10 μm promoted obvious AOX accumulation, but only 0.1 μm or lower ACC concentration had significant effects on tomato drought tolerance. Based on these results, 0.1 μm ACC was used in our subsequent experiments. We found that ACC pretreatment could obviously elevate *V*_t_ and *V*_alt_ during the first 7 days after withholding water, whereas aminoethoxyvinylglycine (AVG, a specific inhibitor of ethylene biosynthesis) pretreated plants showed relative lower *V*_t_ and *V*_alt_ compared with controls (Figure 1e and f). These results demonstrated that AOX might involve in ethylene‐induced drought tolerance.

### AOX is essential in ethylene‐induced tomato drought tolerance

**Figure 2:**
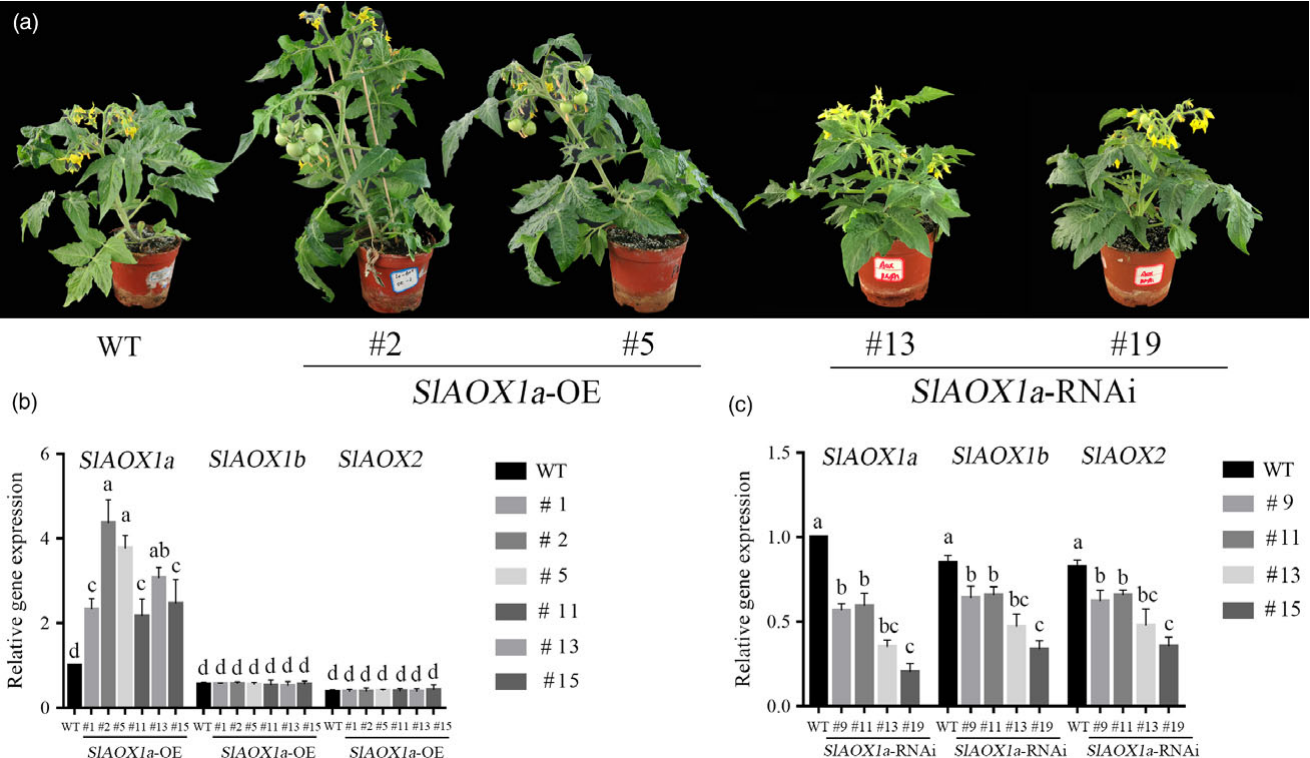
Identification of *SlAOX1a* transgenic lines. (a) Photographs of F2 progeny of transgenic tomato. (b, c) Expression of *SlAOX1a, SlAOX1b* and *SlAOX2* in F2 progeny of transgenic lines. The age of the plants was 6–7 weeks. The data are presented as the means of five biological replicates (±SE). Significant differences (*P* < 0.05) are denoted by different lowercase letters. Three independent experiments were performed with similar results.

To further explore the function of AOX in ethylene‐induced drought tolerance, the transgenic tomato overexpressing *AOX1a* (*35S‐AOX1a‐OE*) and suppressing *AOX* (*AOX1a‐RNAi*) plants were generated. As shown in Figure 2b, all six *35S‐AOX1a* transgenic lines were tested and showed increased levels of *AOX1a*. The *35S*‐*AOX1a*‐*OE‐2* and *35S*‐*AOX1a‐OE‐5* lines showed the greatest expression levels, while *AOX1b*,* AOX1c* and *AOX2* exhibited no changes among these transgenic lines. Among all four *AOX1a‐RNAi* transgenic lines, *aox13* and *aox19* exhibited the most severe *AOX* reduction (90% for the *AOX1a* transcript and ~50% for the *AOX1b* and *AOX2* transcripts) (Figure 2c). The respiration in transgenic plants showed the different changes compared with WT plants (Figure S2). Therefore, 35S‐*AOX1a*‐*OE‐2*, 35S‐*AOX1a*‐*OE‐5*,* aox13* and *aox19* transgenic lines were selected for further experiments (Figure 2a). There were also some differences among these mutants, for example the axillary bud outgrowth and the activity of antioxidant enzymes (Figures S3 and S4).

**Figure 3:**
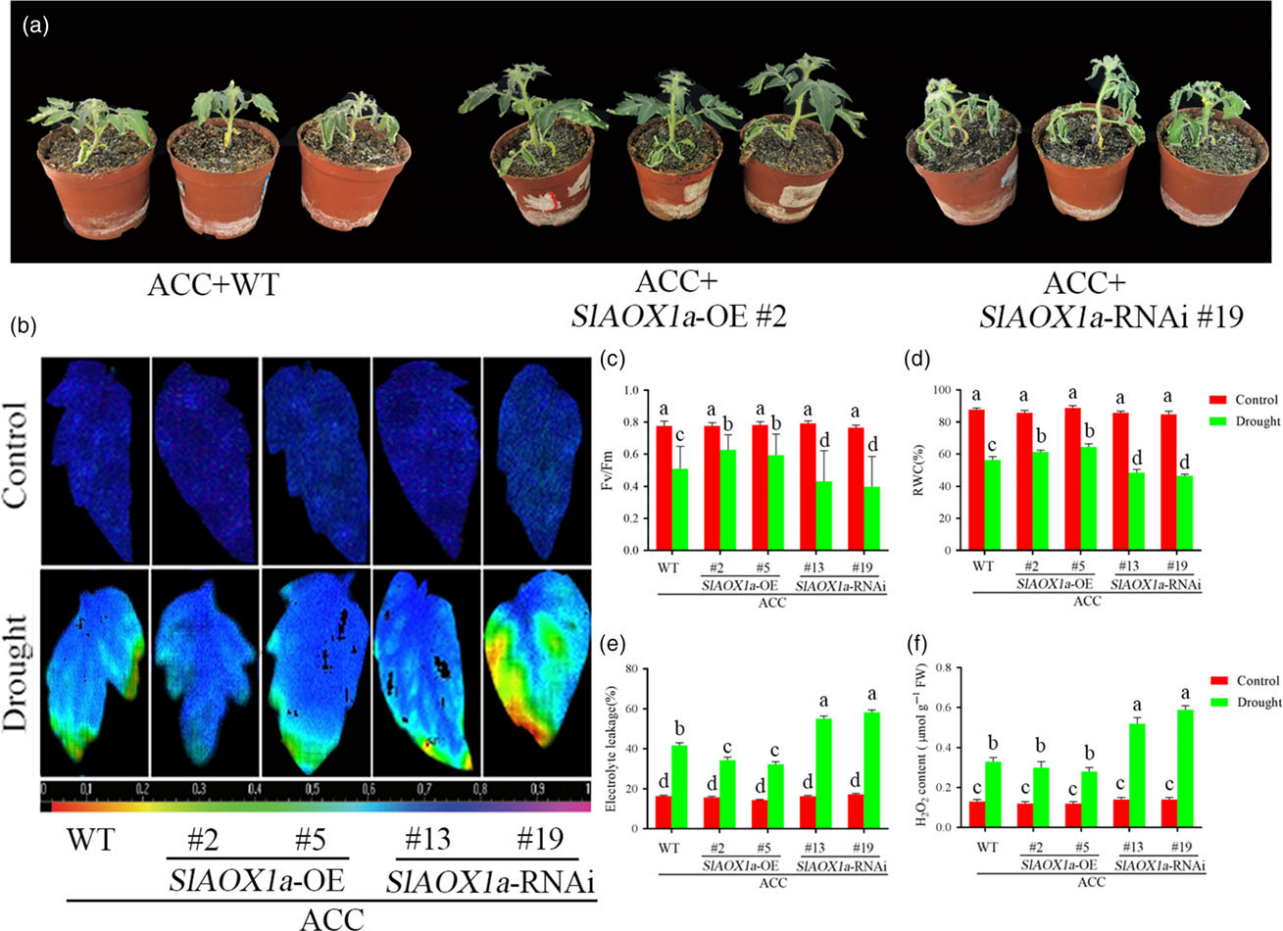
Drought tolerance in ACC‐pretreated transgenic *SlAOX1a* lines. (a) Photographs of ACC‐pretreated WT and transgenic *SlAOX1a* lines in the 14th day under drought stress. (b, c) Changes of the maximum PSII quantum yield (*F*_v_/*F*_m_) in ACC‐pretreated WT and transgenic *SlAOX1a* lines in the 14th day under drought stress. (d‐f) RWC (d), EL (e) and H_2_O_2_ content (f) of ACC‐pretreated WT and transgenic *SlAOX1a* lines in the 14th day under drought stress. Ten plants were used for each treatment group, and a picture of one representative leaf is shown. Bars represent mean and standard deviation of values obtained from six independent plants. Significant differences (*P* < 0.05) are denoted by different lowercase letters.

We further compared the drought tolerance of AOX mutants after pretreated with ACC. Of these, two *AOX1a‐*RNAi lines showed impaired drought tolerance compared with WT plants, as reflected by decreases in the *F*_v_/*F*_m_, relative water content and increases in electrolyte leakage. On the contrary, the *OE‐2* and *OE‐5* plants showed greater drought tolerance compared with WT plants (Figure 3a‐e). Moreover, we found the H_2_O_2_ content, a marker for ROS level which was generated by oxidative damage, was significantly increased by drought and was much higher in *aox13* and *aox19* mutants than WT plants. However, the *35S‐SlAOX1a*‐*OE* plants showed decreased ROS generation under drought (Figure 3f). Therefore, we conclude that a pivotal role of AOX in drought tolerance could be mediated by ethylene.

### Autophagy plays an important role in ethylene‐mediated drought tolerance

**Figure 4:**
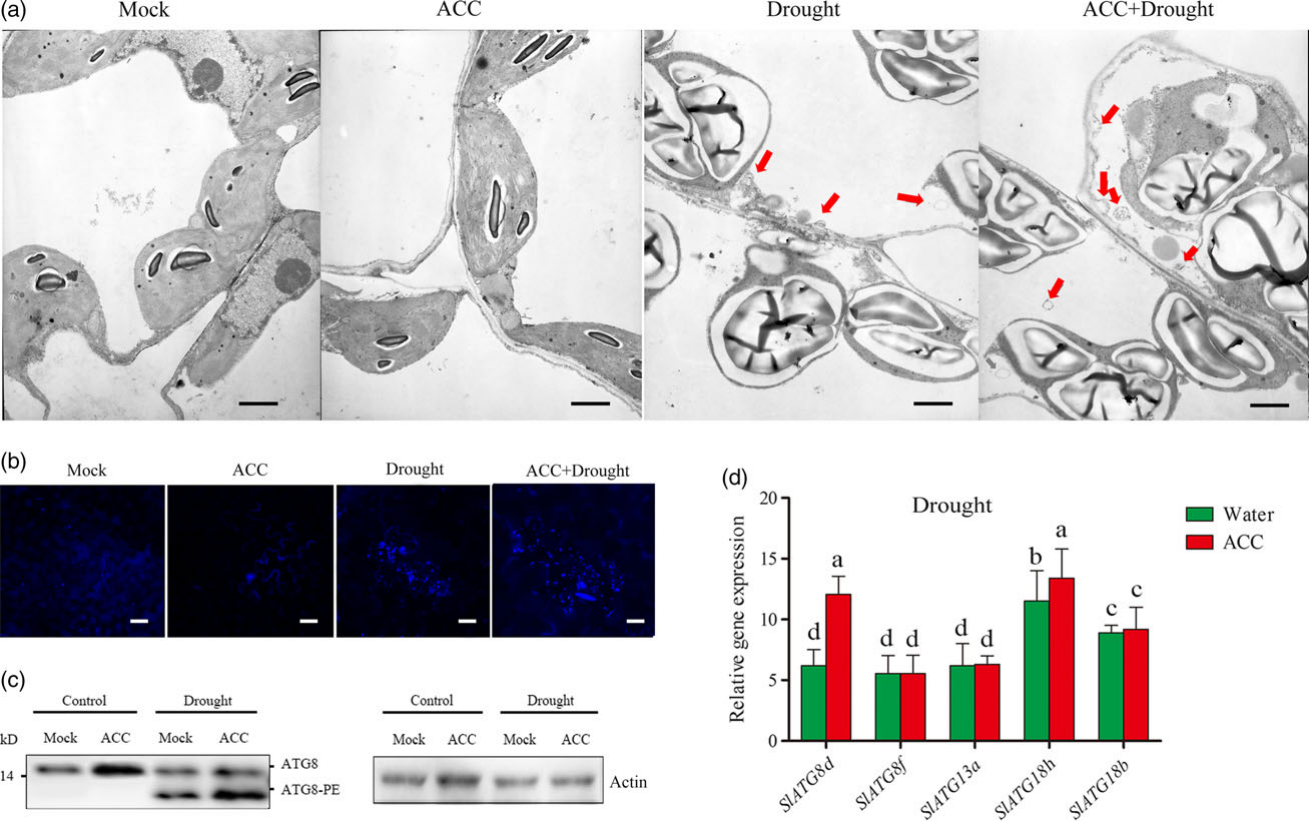
Autophagosomes accumulation in tomato leaves under drought stress. (a) Representative transmission electron microscopy (TEM) images of autophagic structures in the mesophyll cells of different treatment plants. Five‐week‐old plants were exposed to dehydration by withholding water, and the mesophyll cells were visualized in the 7th day by TEM. Autophagic bodies are indicated by red arrows. Bars: 1 μm. (b) MDC‐stained autophagosomes of different treatment groups in the leaves. Five‐week‐old plants were exposed to dehydration by withholding water, and the leaves were MDC‐stained and visualized in the 7th day by fluorescence microscopy. MDC‐labelled structures are shown as blue signals. Bars: 25 μm. (c) ATG8 protein levels in mock and ACC‐pretreated plants after drought treatment. ATG8 and ATG8‐PE are the nonlipidated and lipidated forms of ATG8, respectively. The β‐actin was used as a loading control for the Western blotting analysis. Experiments were repeated three times with similar results. Means with the same letter did not significantly differ at *P* < 0.05 according to the Duncan multiple range tests.

To explore the possible influences caused by ethylene in tomato under drought stress, transmission electron (TEM) was used to monitor the changes of organelles in the leaf cell under drought stress. As shown in Figure 4a, there were little differences between water pretreated and ACC‐pretreated tomatoes under normal condition. Whereas the most intuitive scan was the twisted chloroplast with expanded starch and the collapsed mitochondria under drought condition. In addition, there were many small vesicles (autophagosome, red arrow) occurring inside the cells, especially in ACC‐pretreated tomato under drought condition. Thus, this result indicated that autophagy might be involved in this progress.

Studies have shown that autophagy participated in plant drought tolerance (Liu *et al*., 2009), so whether autophagy participated in ethylene‐mediated drought responses need be further investigated. The monodansylcadaverine (MDC, an autophagosome‐specific autofluorescent dye) was used to directly stain the autophagosome‐like components. Anti‐ATG8 was used to detect the formation of Atg8‐phosphatidylethanolamine (PE) conjugates as a marker for autophagic activation (Wang *et al*., 2015). Under normal water supply conditions, we observed low numbers of punctate fluorescent signals and slight Atg8‐PE band in the plants whatever pretreated with water or ACC. After two‐week drought treatment, either increased numbers of punctate fluorescent signals or faster migration of the Atg8‐PE bands was observed. Importantly, ACC‐pretreated tomato exhibited relative higher autophagy activity than water pretreated tomato under drought (Figure 4b and c). Then, the changes in transcription level among all the *ATG* genes were tested and we found *ATG8d* and *ATG18h* were obviously induced after ACC pretreatment under drought stress (Figure 4d). These results demonstrated that autophagy could be further activated by ethylene under drought.

**Figure 5:**
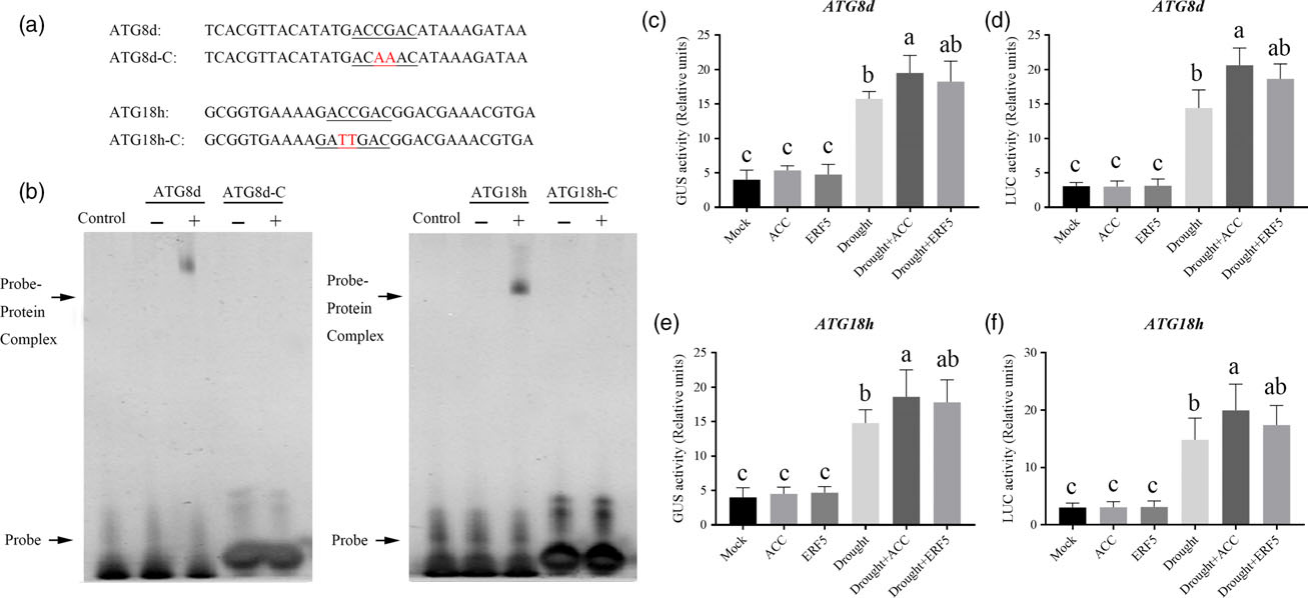
Analysis of *ATG8d* and *ATG18h* promoters. (a) Oligonucleotide used in the electrophoretic mobility shift assays (EMSA). The WT and mutated DRE sequences are underlined. The mutated bases were indicated in red. (b) EMSA showing ERF5 bound to the DRE sequences of the *ATG8d* or *ATG18h* promoters. Recombinant ERF5 was purified from *E. coli* cells and used for DNA binding assays. His was included as the negative control. (c‐f) Relative GUS and LUC activity of *ATG8d* (c,d) and *ATG18h* (e,f) promoters were detected under control or drought condition by different treatments. *N. benthamiana* plants were treated with 0.1 μm ACC for 12 h, and then, the leaves were transiently transformed with different constructs. For drought treatment, the *N. benthamiana* plants were first treated with 0.1 μm ACC for 12 h and then the leaves were transiently transformed with different constructs, after another 12 h, they were treated with 16% PEG6000 for 60 h. The CaMV 35S promoter was fused to GUS or LUC as a control for variation in transformation rate. Bars represent mean and standard deviation of values obtained from three biological repeats. Means with the same letter did not significantly differ at *P* < 0.05 according to the Duncan multiple range tests.

After the investigation of several ethylene response factors (ERFs) which were demonstrated to be induced by drought stress, we found that *SlERF5*, a typical drought responsive transcription factor, was significantly induced by ACC treatment under drought (Figure S5). To examine the possible regulation of tomato *ATG* genes by ERF5, we inspected 1.5‐kb sequences located upstream of the predicted transcriptional start sites of 26 tomato *ATG* genes. Only *ATG8d* and *ATG18h* promoters were shown to contain DRE‐binding site (ACCGAC); therefore, electrophoretic mobility shift assay (EMSA) was performed to explore whether ERF5 could directly bind these promoters (Figure 5a and Figure S10). As Figure 5b shows, probe‐protein complex was detected using *ATG8d* and *ATG18h* promoter probes. When we mutated the core DRE‐binding site of these two genes, as *ATG8d*‐competitor and *ATG18h*‐competitor, the binding to the complexes was vanished. These results suggested that the ERF5 protein specifically binds to the *ATG* sequences in the synthesized probes of the *ATG8d* and *ATG18h* promoters *in vitro*. Transient transfection assay revealed that ACC treatment or ERF5 protein induced the expression of *ATG8d‐pro:LUC* and *ATG18h‐pro:LUC* in *N. benthamiana* as compared to the control under drought stress (Figure 5c‐f).

**Figure 6:**
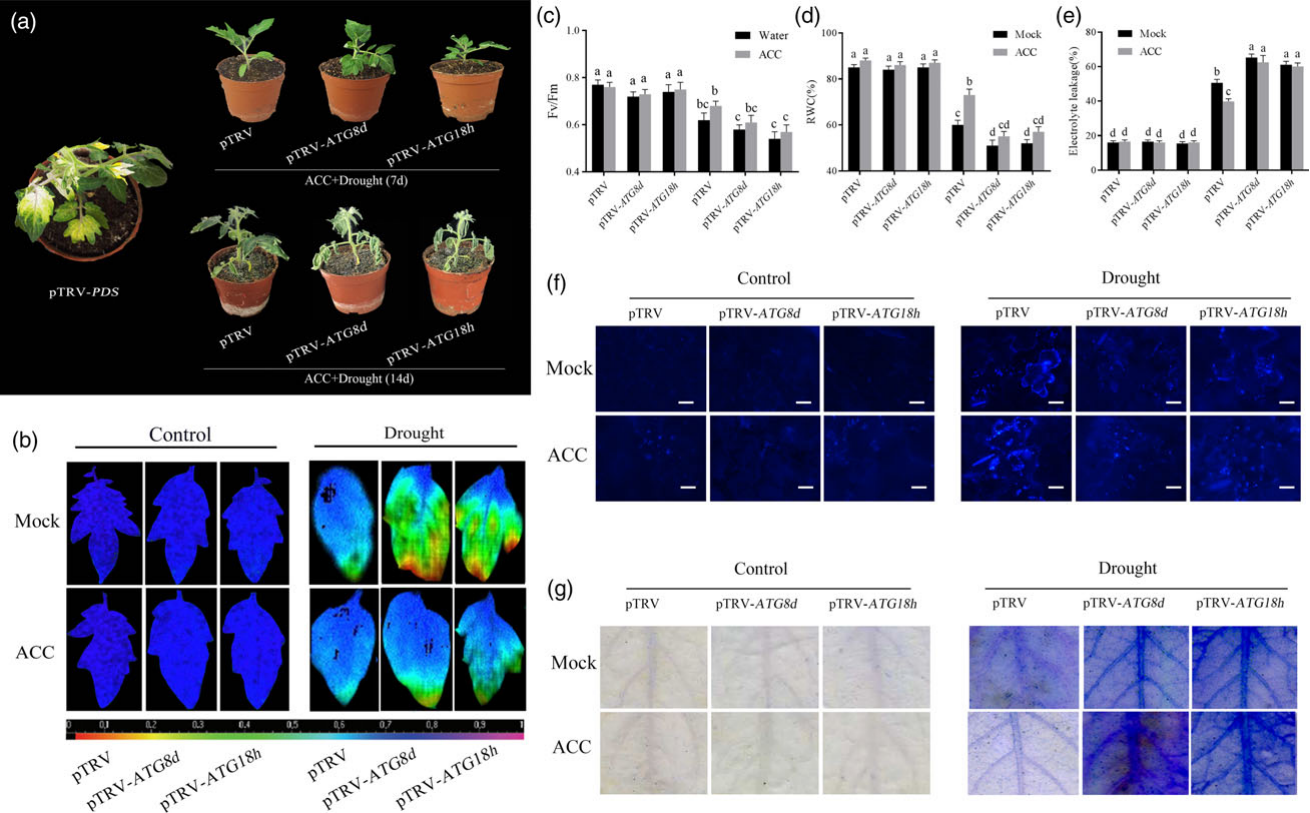
Reduced tomato drought tolerance in TRV‐*ATG8d* and TRV‐*ATG18h* plants. TRV‐*SlPDS* was used as positive control, and TRV‐mock was used as negative control. (a) Phenotypes of ACC‐induced drought tolerance in TRV‐*ATG8d* and TRV‐*ATG18h* plants (7d: upper; 14d: lower). (b, c) Changes of the maximum PSII quantum yield (*F*_v_/*F*_m_) in ACC‐pretreated *ATG8d*‐ and *ATG18h*‐silenced plants in the 14th day under drought conditions. (d, e) Detection of RWC and EL in ACC‐pretreated TRV‐*ATG8d* and TRV‐*ATG18h* plants under different condition. (d) MDC‐stained autophagosomes of different treatment groups in the leaves of TRV, TRV‐*ATG8d,* and TRV‐*ATG18h* plants. Five‐week‐old plants were exposed to dehydration by withholding water, and the leaves were MDC‐stained and visualized in the 7th day by fluorescence microscopy. MDC‐labelled structures are shown as blue signals. Bars: 25 μm. (e) Trypan blue staining showing cell death in the leaves of TRV, TRV‐*ATG8d* and TRV‐*ATG18h* plants. Means with the same letter did not significantly differ at *P* < 0.05 according to Duncan multiple range tests. Three independent experiments were performed with similar results.

To investigate the roles of these two *ATG* genes act in ethylene‐induced drought tolerance, the method of virus‐induced gene silencing (VIGS) was used to generate *ATG8d*‐/*ATG18h*‐silenced plants and the results showed that the levels of these genes were only 30%–40% of the TRV control plants (Figure S6). After pretreated with ACC, *ATG8d*‐ and *ATG18h*‐silenced plants showed severe damage compared with ACC‐pretreated TRV control plants under drought stress (Figure 6a). Figure 6b and c showed that the photosynthetic efficiency in ACC‐pretreated TRV‐*ATG8d* and TRV‐*ATG18h* plants was obviously lower than TRV control plants under drought stress, which indicated the *ATG8d* and *ATG18h* are essential in ethylene‐induced drought resistance. The RWC and EL in ACC‐pretreated *ATG8d*‐ and *ATG18h*‐silenced plants further confirmed above result (Figure 6d and e). As shown in Figures 6f and Figure S7, autophagy activity significantly decreased in *ATG8d*‐ and *ATG18h*‐silenced plants, with or without ACC pretreatment. Furthermore, we found that *ATG8d*‐ and *ATG18h*‐silenced tomato leaf cells were earlier to be death compared with control plants under drought stress (Figure 6g), which means that autophagy is indispensable and functioning to prevent soil water deficit‐triggered rapid cell death.

### The collaboration of AOX and autophagy are indispensable in ethylene‐mediated drought tolerance

**Figure 7:**
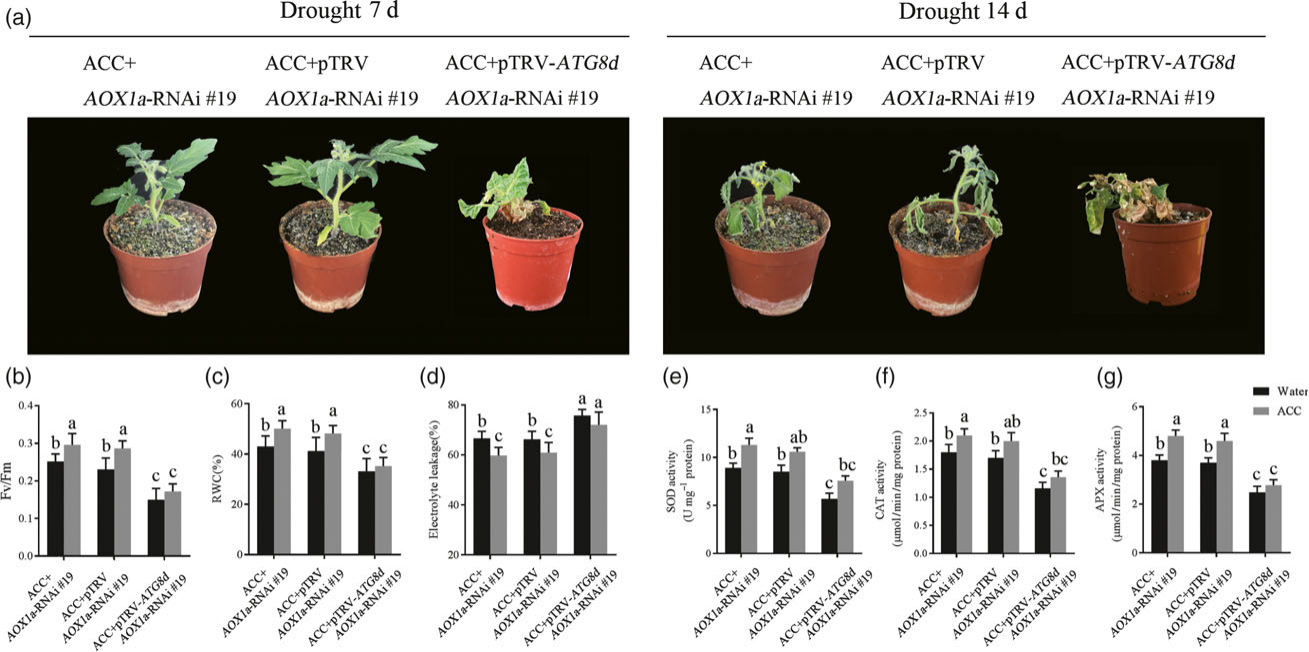
The indispensable roles of AOX and autophagy in ethylene‐mediated drought tolerance. (a) Photographs of ACC‐pretreated TRV and TRV‐*ATG8d* in *aox19* plants under drought. (b‐d) The *F*_v_/*F*_m_(b), RWC(c) and EL(d) in different groups under drought stress. (e‐g) The antioxidant enzyme activity of SOD (e), CAT (f) and APX (g) in different groups under drought stress. Means with the same letter did not significantly differ at *P* < 0.05 according to Duncan multiple range tests. Three independent experiments were performed with similar results.

As either AOX or autophagy participated in ethylene‐induced drought resistance, we next investigated whether the increased AOX and autophagy capacity are both essential in this process. We used VIGS technical to silence *ATG8d* in aox*19* mutant plants for generating *ATG8d‐* and *AOX*‐co‐silenced plants. The silence efficiency was confirmed by RT‐PCR (Figure S8). As shown in Figure 7a, even at the 7th day after withholding water, the *ATG8d‐* and *AOX*‐co‐silenced plants exhibited badly growth condition, falling leaves, dwarf, wilting shoots. Thus, the damage level among these mutants were detected and we found that the *F*_v_/*F*_m_ and RWC were significant lower whereas EL was higher in *ATG8d‐* and *AOX*‐co‐silenced plants compared to *aox19* and TRV control *aox19* plants with or without ACC pretreatment, especially in drought stress (Figure 7b‐d). That means the *ATG8d‐* and *AOX*‐co‐silenced plants were severely damaged under drought stress. Several antioxidant enzymes activity were almost collapse in *ATG8d‐* and *AOX*‐co‐silenced plants under drought stress (Figure 7e‐g). Notably, ACC pretreatment actually alleviated the defence systems reduction. These results showed that AOX and autophagy both play positive and indispensable roles in ethylene‐induced tomato drought tolerance.

### The relationship between AOX and autophagy in ethylene‐induced drought tolerance

**Figure 8:**
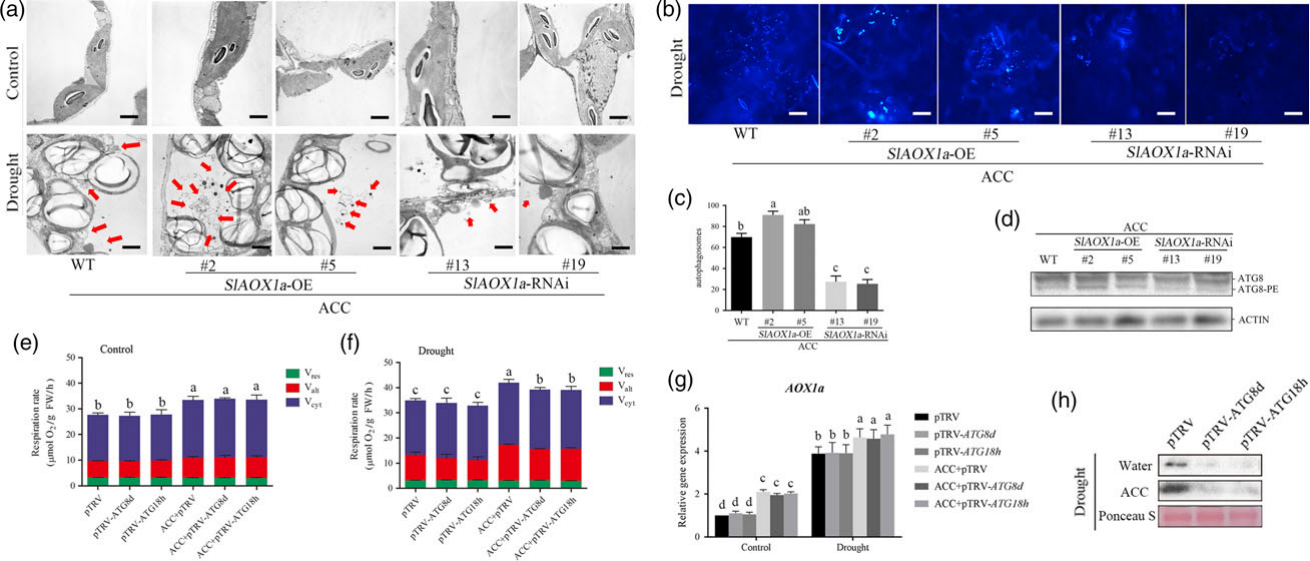
AOX and autophagy worked collaboratively in ethylene‐induced drought tolerance. (a) Representative transmission electron microscopy (TEM) images of autophagic structures in the mesophyll cells of different treatment in transgenic plants. Autophagic bodies are indicated by red arrows. Bars: 1 μm. (b‐d) MDC staining (b), autophagic activity(c) and ATG8 protein levels (d) for autophagosomes, respectively. Bars: 25 μm. (e‐h) Detection of respiration rate (e, f), *AOX1a* expression level (g) and AOX protein level (h) in ACC‐pretreated TRV‐*ATG8d* and TRV‐*ATG18h* plants. Ponceau S‐stained membranes are shown below the blots to indicate the amount of protein loaded per lane. Means with the same letter did not significantly differ at *P* < 0.05 according to Duncan multiple range tests. Three independent experiments were performed with similar results.

**Figure 9:**
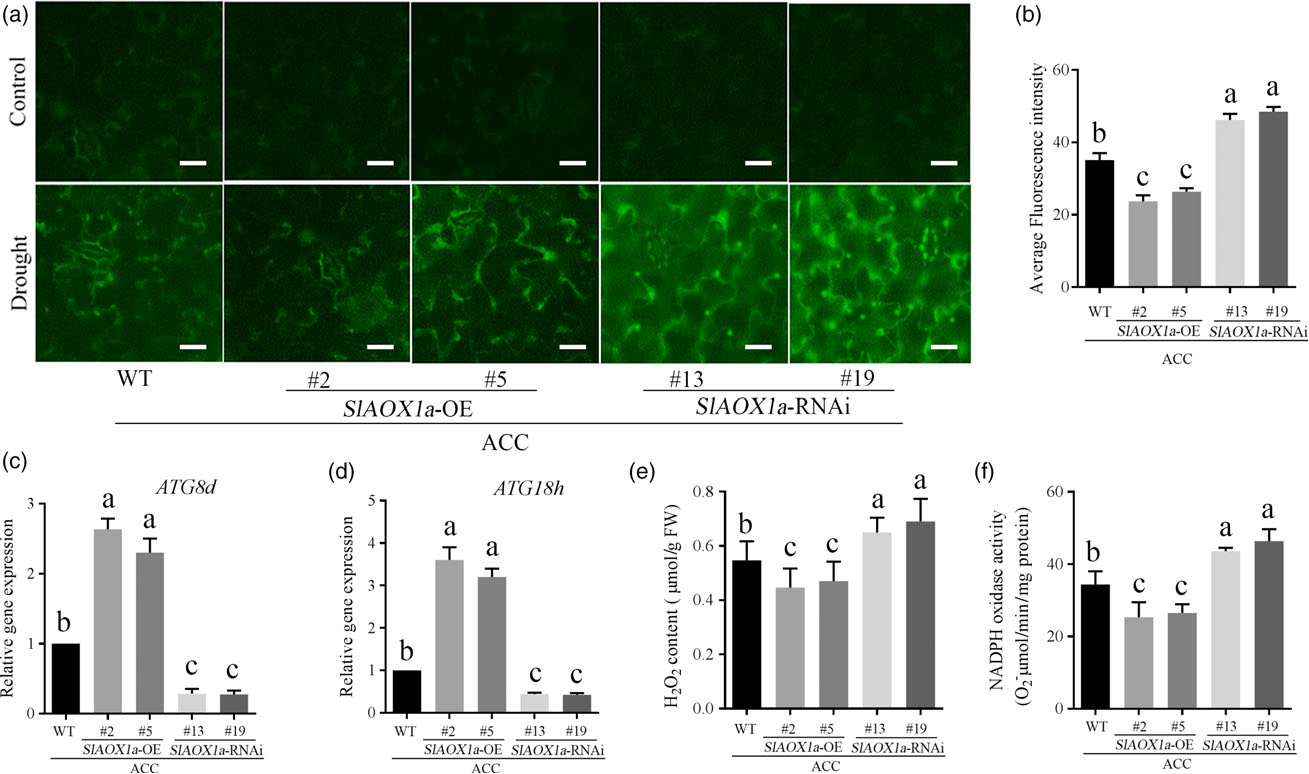
Quantitative measurements of H_2_O_2_ and autophagic capacity. (a, b) H_2_ DCF‐DA staining showing ROS accumulation in the leaves of different treatment groups in the 14th day under drought stress. (c, d) *ATG8d* (c) and *ATG18h* (d) expression level in ACC‐pretreated transgenic tomatoes in the 14th day under drought stress. (e, f) H_2_O_2_ content and (e) NADPH activity (f) in ACC‐pretreated transgenic tomatoes in the 14th day under drought stress. Means with the same letter did not significantly differ at *P* < 0.05 according to Duncan multiple range tests. Three independent experiments were performed with similar results.

To monitor the changes of autophagy activity in ACC‐pretreated AOX transgenic tomato, we used transmission electron microscopy (TEM), MDC and anti‐ATG8 to survey the autophagy capacity. As was shown in Figure 8a‐d, ACC‐pretreated *OE‐2* and *OE‐5* plants exhibited higher whereas *aox13* and *aox19* exhibited lower autophagic activity than WT plants. The TEM detection showed that the starch in the chloroplasts was inflated and almost occupied most chloroplast space under drought condition. The mitochondrial were also almost collapsed because there were almost no integrity mitochondria structures were found in *aox* mutants. To figure out the crosstalk between AOX and autophagy, we then checked the AOX capacity in ACC‐pretreated TRV‐*ATG8d* and TRV‐*ATG18h* plants. The total respiration rate (*V*_t_) and alternative pathway respiration rate (*V*_alt_) had no differences between TRV‐*ATG8d* or TRV‐*ATG18h* and TRV control plants with or without ACC pretreatment under normal condition (Figure 8e and f). However, *V*_t_ and *V*_alt_ were significant lower in *ATG8d*‐ and *ATG18h*‐silenced plants compared with TRV control plants under drought stress. Furthermore, although ACC pretreatment elevated *V*_t_ and *V*_alt_ under drought, silencing *ATG8d* and *ATG18h* alleviated the increasing level of *V*_t_ and *V*_alt_ (Figure 8e and f). Then, we examined the transcription level of *AOX1a* and there were no significant changes between two *ATG*‐silenced plants and TRV control plants (Figure 8g). To explore the reason why ethylene‐induced *V*_alt_ was almost vanished in TRV‐*ATG8d* and TRV‐*ATG18h* plants under drought, we analysed the AOX protein level in these two *ATG*‐silenced plants. As shown in Figure 8h, AOX protein level was significantly decreased in *ATG8d*‐ and *ATG18h*‐silenced plants. So we presumed that the compromised *V*_alt_ was probably due to the AOX protein degradation under drought stress. Previous studies have reported that autophagy could help to recycle any molecules that are useful for the cellular components, which might be the reason to explain why the AOX protein level was obvious lower when *ATG8d*/*ATG18h* was silenced under drought stress. In accordance with previous researches, the ROS level was higher in AOX‐silenced plants whereas lower in AOX‐overexpressing plants, it could be result from the AOX functioning to scavenge ROS (Moore and Albury, 2008; Xu *et al*., 2012b) and play a role in the process of programmed cell death (PCD) in plants (Vanlerberghe *et al*., 2002; Vanlerberghe *et al.,* 2009; Zhang *et al*., 2014a). To make it clear, we compared the ROS generation and autophagic activity in different ACC‐pretreated AOX transgenic plants under drought stress. H_2_DCF‐DA fluorescence probe was used to detect the H_2_O_2_ generation. As Figure 9a and b shown, the ROS accumulation decreased in *OE‐2* and *OE‐5* plants whereas elevated in *aox13* and *aox19* plants when compared with the control plants. In contrast, the autophagic activity in *OE‐2* and *OE‐5* showed increased level but decreased in *aox13* or *aox19* plants when compared with the control plants.

To confirm above results, we further checked the H_2_O_2_ content, the most stable type of ROS, NADPH oxidase activity which is a crucial source of H_2_O_2_ generation and the two key *ATG* genes transcription in ACC‐pretreated transgenic plants under drought stress condition. Consistent with previous results, *AOX1a*‐overexpressing tomato exhibited lower H_2_O_2_ accumulation and higher expression level of autophagy than WT plants. On the contrary, *AOX1a‐RNAi* plants showed relative higher H_2_O_2_ generation and extremely weak autophagy capacity compared with WT plants (Figure 9c‐f). These results demonstrated that both AOX‐mediated ROS signalling and autophagy might be involved in ACC‐mediated drought responses.

### AOX‐dependent ROS signalling is a key factor for the induction of autophagy in response to drought stress

**Figure 10:**
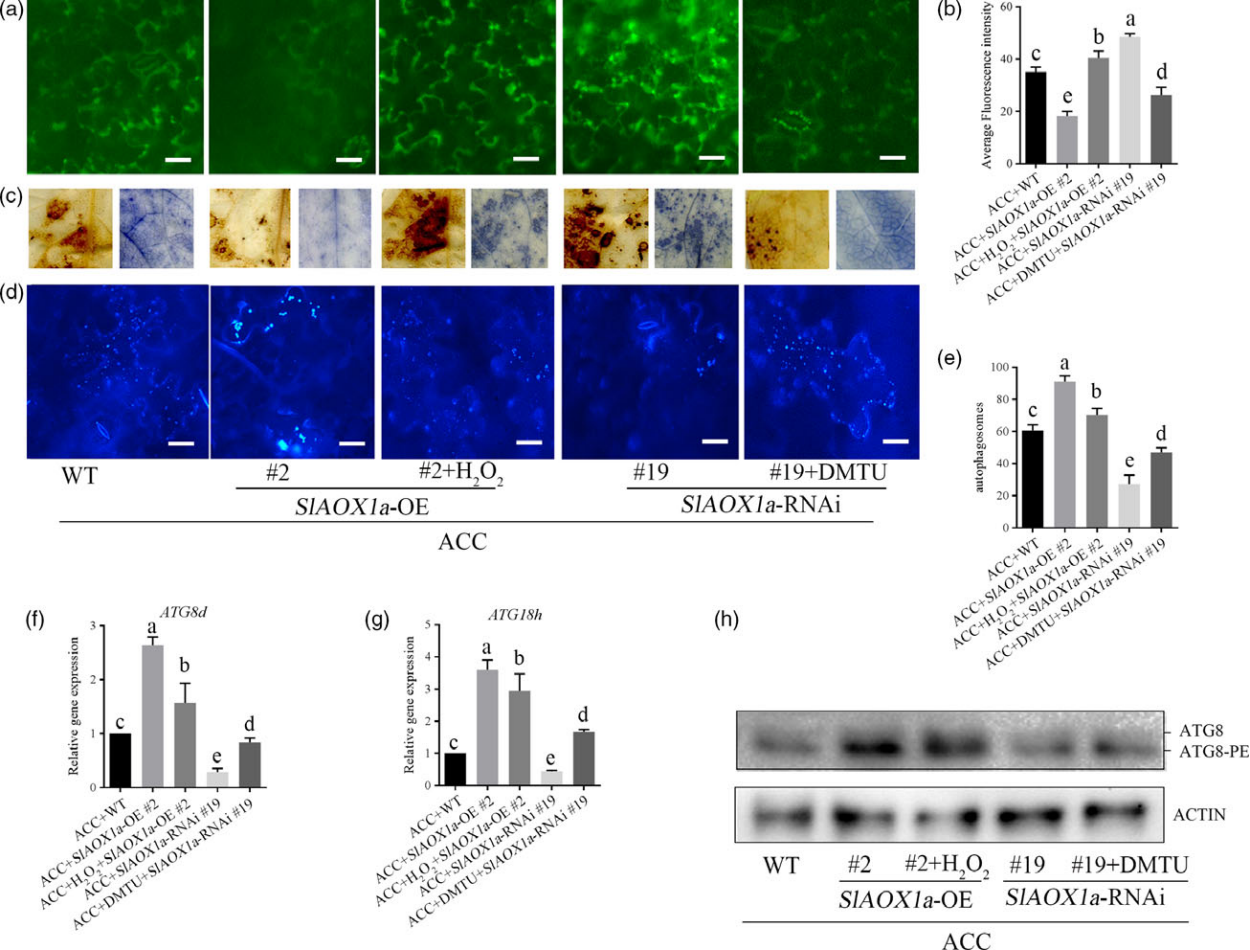
Changing ROS level contributing to different autophagic capacity. (a‐c) In *vivo* and *vitro* detection of H_2_O_2_ level in the 14th day under drought stress. (d‐e) MDC staining (d) and autophagic activity (e) in different treatments of transgenic tomatoes in the 14th day under drought condition. (f‐h) *ATG8d* (c), *ATG18h* (d) and ATG8 protein levels expression level in different treatments of transgenic tomatoes in the 14th day under drought condition. ATG8 and ATG8‐PE are the nonlipidated and lipidated forms of ATG8, respectively. The β‐actin was used as a loading control for the Western blotting analysis. Exogenous H_2_O_2_ at or DMTU was sprayed onto tomato plants every 3 d under drought condition. Means with the same letter did not significantly differ at *P* < 0.05 according to Duncan multiple range tests. Three independent experiments were performed with similar results.

To further elucidate the underlying crosstalk between AOX‐mediated ROS signalling and autophagy in ACC‐mediated drought response, we measured H_2_O_2_ and autophagic activity in *OE‐2* and *aox19* plants with different pharmacological treatments. In *OE‐2* plants, foliar application of H_2_O_2_ enhanced ROS signalling accompanying with a decreased autophagic activity under drought in response to ACC. In *aox19* plants, the dimethylthiourea (DMTU, a H_2_O_2_ scavenger) treatment compromised ROS generation whereas increased the autophagic activity (Figure 10a‐c). In accordance with ROS fluorescence, the H_2_O_2_ content and NADPH activity were relative higher when applied with exogenous H_2_O_2_ compared with water control treatment (Figure S9). By the way, DMTU treatment could scavenge the H_2_O_2_ generation but enhanced autophagic activity in ACC‐pretreated *aox19* plants (Figure 10d‐h). We found the autophagic activity was higher when the H_2_O_2_ level was keeping in a relative low level in these samples have been improved in AOX capacity. Therefore, it seems that the autophagy could be triggered by the relative low ROS signal and function in plants against drought stress (Figure S9).

## Discussion

Drought stress has been well studied and known as a range of separate stresses, such as high vapour pressure deficit and soil water deficit, often associated with oxidative stress, decreased soil nutrient availability, increasing soil salinity and mechanical impedance to root growth in hard soil. It is widely known that ethylene played an important role in the processes of stomatal control and root elongation (Sharp and LeNoble, 2002; Tanaka *et al*., 2005). As shown in Figure S1, 0.1 μm ACC pretreatment could significantly enhance tomato drought resistance. It is worth mentioning that Desikan *et al*. (2006) reported that ethylene could induce stomatal closure via *AtRBOHF*‐mediated hydrogen peroxide synthesis in *Arabidopsis;* therefore, we monitored the stomata conductance between ACC and AVG treatments. The stomatal aperture in ACC‐pretreated tomato leaves was lower compared with controls whereas AVG treatment compromised this process in the 7th day under normal or under drought condition (Figure S1). Studies also showed that ethylene production in various plant tissues are enhanced when plants were challenged by various types of stresses (Morgan and Drew, 1997). Recent studies showed ethylene‐insensitive mutants exhibited improved drought tolerance in Arabidopsis and Maize (Shi *et al*., 2015). Previous studies have also shown that inhibition of ethylene‐induced maturation led to an obvious impaired drought tolerance and reduction in carbohydrate consumption, grain‐filling and spikelet fertility in rice (Du *et al*., 2014). Shi *et al*., 2006 had reported the dual role of ethylene concentration in regulating cotton fibre growth. It must also be noted, although ethylene can promote biomass accumulation, it also has a strong effect to reduce extension growth, when plants faced to stress condition (Gallie *et al*., 2009). Several ethylene responsive factors (ERFs) could also enhance drought tolerance in tomato and tobacco (Pan *et al*., 2012; Quan *et al*., 2010). So, combining with former reports, we speculated that the mechanism of ethylene‐induced drought tolerance is the so‐called giving up a rook to save the king. Specifically, it reflected in the following aspects: shedding of older leaves, diversion from vegetative to reproductive growth, accumulation of osmotically active solutes involved in the maintenance of cell turgor, and the synthesis of antioxidant proteins (Chaves, Maroco & Pereira 2003; Wilkinson, & Davies 2010). It is well known that mitochondrial CN‐resistant alternative pathway plays a pivotal role in plant environmental stresses resistance, as AOX located in the mitochondrial inner membrane and acts as a terminal oxidase in the plant mitochondrial electron transport chain; thus, it functions in the releasing of excess energy as heat (Millenaar and Lambers, 2003) or serve a general function by limiting mitochondrial ROS formation (Bartoli *et al*., 2005; Kühn *et al*., 2015; Wang *et al*., 2012). Studies also showed that ethylene could induce AOX capacity in response to abiotic stresses (Wang *et al*., 2010, 2012). 1‐(Aminocarbonyl)‐1‐cyclopropanecarboxylic acid (ACC) is the precursor of ethylene, which is catalysed by ACC oxidase (ACO). When ACC converted to ethylene,the coproduction of this reaction was cyanide (CN) which could trigger the generation of ROS or H_2_O_2_, thereby enhance alternative respiration and AOX capacity (Miyazaki and Yang, 1987; Siedow and Berthold, 1986). CN‐triggered ROS or H_2_O_2_ was the signal molecule which could activate the ROS‐responsive motifs in *AOX1* promoter (Deng *et al*., 2015; Xu *et al*., 2012b). Therefore, the relative lower concentration of ACC could generate lower concentration of CN and H_2_O_2_ which promote *AOX* expression. *AOX1a* expression was increasing as the ACC concentration rising, but 10μM ACC showed similar effect to 1 μm ACC indicating that the AOX was sensitive to ACC which reached the maximum capacity when ACC concentration is 1 μm. It is well known that higher CN is extreme toxic to all living cells. Science ACC would induce CN production, as the above mentioned, it was not hard to imagine that higher concentration of ACC pretreatment decreased the plants drought tolerance. Accordingly, the dual role of ACC might be due to the amount of CN and H_2_O_2_ which produced with ethylene synthesis (Deng *et al*., 2015; Ho *et al*., 2008; Xu *et al*., 2012b). Based on the results presented in this study, we suppose that AOX is definitely participated in ethylene‐induced drought tolerance. Interestingly, we found that F2 progeny of *AOX1a‐RNAi* tomato seedlings exhibited more lateral shoots growth and less H_2_O_2_ accumulation than wild‐type tomato seedlings, whereas conversely in *35S‐AOX1a*‐OE plants (Figure S3). As Chen *et al*. (2016) reported that H_2_O_2_ plays a critical role in axillary bud outgrowth in tomato plants, we speculated that the changing AOX capacity leads to altering H_2_O_2_ accumulation and thus results in the variation of lateral bud development. Surprisingly, the H_2_O_2_ content in *AOX1a‐RNAi* was relative lower than WT and *AOX1a*‐OE plants under normal growth condition. As previous studies have reported that the H_2_O_2_ concentration is governed by either synthesis or degradation, and knockdown of AOX could active multiple H_2_O_2_‐scavenging systems under normal growth conditions (Amirsadeghi *et al*., 2006; Giraud *et al*., 2008). In tobacco, this may overcompensate for the lack of AOX, in terms of H_2_O_2_ scavenging (Amirsadeghi *et al*., 2006; Cvetkovska *et al*., 2014). Conversely, overexpression of AOX in tobacco can suppress H_2_O_2_‐scavenging systems, resulting in elevated H_2_O_2_ (Pasqualini *et al*., 2007). So this trait of the AOX transgenic tomato is consistent with the previously researches. The results showed that *SlAOX1a*‐RNAi lines have suppressed *SlAOX1b* and *SlAOX2* expressions (Figure 2c). It might due to the homologous sequence of the *SlAOX1a*,* SlAOX1b* and *SlAOX2* cDNA fragment which we chose to generate a double‐stranded RNA interference (RNAi) trial to knock down the AOX capacity thereby to repress alternative respiration pathway. Furthermore, the *SlAOX1a*‐RNAi lines which we used may avoid the function redundancy of other *SlAOX* gene members. As ACC+*SlAOX1a*‐RNAi lines exhibited decreased drought tolerance whereas ACC+*35S*‐*SlAOX1a‐OE* lines showed enhanced drought tolerance compared with ACC+WT plants, it demonstrated that modification of *SlAOX1a* influenced AOX capacity thereby affected drought tolerance (Figure 3). Giraud *et al*., 2008 reported that AOX is necessary for drought response and the absence of *AtAOX1a* results in acute sensitivity to combined light and drought stress. Overexpression of *AOX1a* could enhance plant tolerance to aluminium phytotoxicity (Liu *et al*., 2014). A large number of studies indicated the indispensible role of AOX in plant drought tolerance (Bartoli *et al*., 2005; Dahal and Vanlerberghe, 2017; Dahal *et al*., 2014). So combined with these results, we can speculate the important role of AOX in plant response to drought. So we can conclude that the drought tolerance changes were caused by *SlAOX1a* together with its two homologs *SlAOX1b* and *SlAOX2*. Our results confirmed that ACC treatment could induce tomato resistance to drought, and AOX is involved in this progress. Furthermore, overexpressing of *AOX1a* alleviate drought‐induced H_2_O_2_ generation thereby amplified ACC‐induced drought tolerance (Figure 3f). Evidences showed that autophagy could regulate plant senescence or starvation‐induced chlorosis (Hanaoka *et al*., 2002; Liu *et al*., 2005). We found ACC pretreatment enhanced autophagy activity via up‐regulating the transcription level of *ATG8d* and *ATG18h* under drought condition whereas had little effect on autophagy under normal growth condition. Previous reports have indicated that ABA‐dependent pathway as well as ABA‐independent, but DREB2‐dependent pathway was essential in plants drought responses (Liu *et al.,* 2009). Ethylene response factors (ERFs) have been shown to bind to DRE‐elements (ACCGAC) and to play a regulatory role in plant responses to abiotic stresses (Cheng *et al*., 2013). Therefore, several ERFs which respond to drought and ACC were examined. *SlERF5*, encoding a typical class III group of ERFs, is induced by abiotic stress (Figure S2). After analysing the promoters of each *ATG* genes, we found there was a conserved DRE‐binding sequence located in the promoters of *SlATG8d* and *SlATG18h*. Promoter analysis showed that these *ATG* genes might only be regulated by ethylene or ERF5 in drought condition. Thus, we speculate that ethylene pretreatment enhanced autophagy in drought probably due to the enhanced transcription activity of *SlERF5*. Therefore, the enhanced ERF5 induced the transcription of these two ATG genes to stimulate autophagy. Previous studies have demonstrated that autophagy is essential in leaf senescence (Hanaoka *et al.,* 2002; Wada *et al*., 2009). Ethylene has been shown to be a regulator of leaf senescence in many plants (Grbić and Bleecker, 1995; John *et al*., 1995). However, there were few reports about ethylene and autophagy in tomato drought responses. These results showed that autophagy was not only enhanced by exogenous ethylene under drought condition but also played a critical role in ethylene‐induced tomato drought tolerance. ACC‐pretreated *SlATG8d* and *SlATG18h* knockdown plants exhibited impaired tolerance and significant cell death, accompanied by decreased photosynthetic efficiency and severe oxidative damage in tomato under drought condition. It means lacking of autophagy led to rapid cell death, indicating that autophagy plays an important role in ethylene‐induced drought resistance. To further explore the function of AOX and autophagy in ethylene‐induced drought tolerance, we knock down the *SlATG8d* in ACC‐pretreated WT and *aox19* plants, both *SlATG8d*‐silenced *aox19* plants exhibited serious wilting after 13d of drought stress. The ACC‐pretreated *SlATG8d*‐silenced *aox19* plants showed the most compromised drought tolerance compared with ACC‐pretreated TRV control plants, not only reflected in the physiological parameter but also exhibited in the rapid death of elder leaves (Figure 7). These results indicating that AOX and autophagy are both necessary and play positive roles in tomato antagonizing drought. Studies have shown that AOX help to scavenge the redundant ROS in response to abiotic stress and plant senescence (Munné‐Bosch and Alegre, 2004; Xu *et al*., 2012b). There were other evidences demonstrating that autophagy played a critical role in plant drought tolerance and mitochondria generation of ROS is a trigger for autophagy (Chen and Gibson, 2008; Lee *et al*., 2012; Liu *et al*., 2009; Minibayeva *et al*., 2012). AOX protein level was obviously decreased when *ATG8d* and *ATG18h* were silenced whereas *AOX1a* transcription level has no changed (Figure 8g and h). Combined with the changing of AOX‐dependent H_2_O_2_ level and autophagic activity in ACC‐pretreated AOX transgenic plants under drought, these results means AOX and autophagy worked collaboratively in response to drought. To further determine the result, exogenous H_2_O_2_ and its scavenger DMTU were used to modify AOX‐dependent ROS signalling. As shown in Figure 10, ACC+H_2_O_2_ pretreatment in *OE‐2* plants compromised the autophagy activity compared with ACC pretreatment in *OE‐2* plants. However, ACC+DMTU pretreatment in *aox19* plants exhibited increased autophagy activity. These results further confirmed that AOX‐dependent ROS signalling modified autophagy participated in ethylene‐induced drought tolerance.

**Figure 11:**
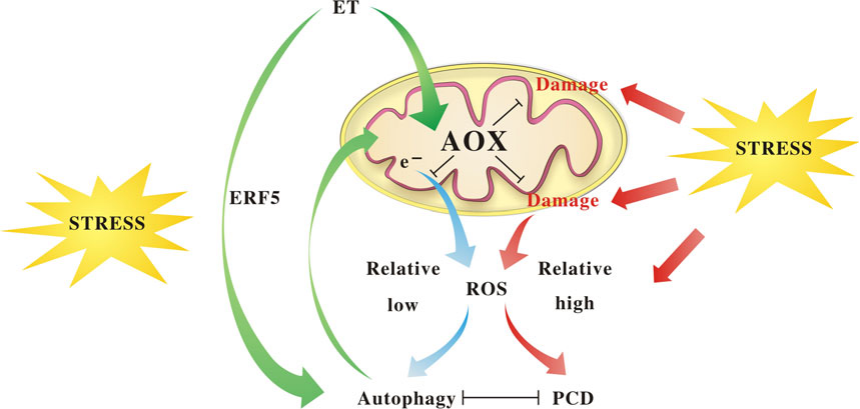
Proposed model for ethylene‐mediated drought tolerance.

In conclusion, it is not difficult to imagine that drought stress led to a gradual degradation of mitochondria and the AOX function as a ROS scavenger to avoid rapid O_2_^‐^ burst while the relative lower ROS level acts as a signal to trigger autophagy. Furthermore, the activated autophagy which may be also regulated by DRE‐binding elements (e.g. ERF5) delayed the processes of mitochondria collapse, thus maintaining the AOX integrity under drought condition. ERF5 might be a key regulator of autophagy which could be stimulated by ethylene under drought condition. The collaborative relationship between AOX and autophagy played critical roles in ethylene‐induced resistance to drought and led to significant survival of plants under stress conditions (Figure 11).

## Experimental procedures

### Plant materials and chemicals

The tomato *Solanum lycopersicum* cv. Micro Tom genotype was used in all experiments. Seeds were germinated and grown in plastic pots filled with a mixture of peat and vermiculite (2:1, v: v). The plants were grown in a controlled growth chamber at 25 °C under 16 h light/8 h darkness and watered daily. The seedlings used in the experiments were 4–5 weeks old.

To induce drought stress, plants in soil were watered to saturation with Hoagland solution or Hoagland plus 0–10 μm ACC and then exposed to dehydration by withholding water for 14 d.

ACC, AVG, H_2_O_2_ and DMTU were purchased from Sigma‐Aldrich (http://www.sigmaaldrich.com). The concentrations used are as follows: ACC, 0.1 μm; AVG, 50 μm; H_2_O_2_, 500 μm; DMTU, 500 μm. Distilled water containing 0.02% v/v Tween‐20 was used as a control treatment.

### Generation and selection of transgenic plants

To generate the tomato overexpression *SlAOX1a* vector, the full‐length coding DNA sequence (CDS) of 1254 bp PCR product was cloned into the pBI121 vector (Clontech, Palo Alto, CA, USA). The homologous sequence of the *SlAOX1a*,* SlAOX1b* and *SlAOX2* cDNA fragment was used for a double‐stranded RNA interference (RNAi) trial. The fragment and its inverted repeat fragment were inserted downstream of the CaMV 35S promoter at the BamHI and SacI restriction sites of the modified PBI121. The construct *AOX1a*‐RNAi was thus generated. The primers were shown in Table S1.

Transgenic plants were generated by *Agrobacterium tumefaciens* (strain EHA105)‐mediated transformation according to the method described previously (Xu *et al*., 2012b), and transformed lines were first selected for kanamycin (70 mg/L) resistance and then analysed by PCR to determine the presence of T‐DNA. The primers designed for NPTII (Kana resistance) were used as marker of PBI121 for identified of transgenic tomato (Table S1).

### VIGS constructs and Agrobacterium‐mediated virus infection

TRV VIGS constructs were used to silence the tomato *PDS*,* ATG8d* and *ATG18h* genes. These genes were generated by PCR amplification using gene‐specific primers (Table S1), digested with the appropriate restriction enzymes and ligated into the same sites in TRV2. The constructs were transformed into *Agrobacterium tumefaciens* strain GV3101. The VIGS assay was performed as described previously (Zhu *et al*., 2016b).

### Oxidative damage estimation

Leaf relative water content (RWC), electrolyte leakage (EL) and trypan blue staining were conducted as described (Zhu *et al*., 2016b).

### Protein extraction and Western blotting analysis

Total proteins were extracted using extraction buffer (50 mm Tris/Cl, pH 6.8, 5% mercaptoethanol, 10% glycerol, 4% SDS and 4 m urea). For Western blotting analysis, approximately 10 μg of protein from each sample was subjected to SDS‐PAGE and transferred to nitrocellulose membranes. Then, the membranes were hybridized with antibody against the AOX protein (Agrisera, cat. No. AS04 054; 1:1000). Specific anti‐ATG8 (Abcam, cat. No. ab77003, 1:1000) was used in the protein blotting analysis. Anti‐β‐actin (Invitrogen cat. No. MA5‐15739, 1:10000) was used as loading control.

### Respiration and chlorophyll fluorescence measurements

Respiratory rate and chlorophyll fluorescence were measured using Clark‐type electrodes (Hansatech, King's Lynn, UK) and an imaging pulse amplitude‐modulated fluorometer (IMAG‐MINI, Heinz Walz, Germany) according to Zhu *et al*. (2016a,b).

### RNA extraction, RT‐PCR and quantitative real‐time PCR

Total RNA was purified from tomato leaves. RT‐PCR and real‐time PCR analyses were conducted as described (Zhu *et al*., 2016a,b). The primer sequences are shown in Table S1.

### MDC staining

To visualize the accumulation of autophagosomes, tomato leaves were excised and then immediately vacuum infiltrated with 100 mm MDC (Sigma‐Aldrich) for 30 min, followed by two washes with PBS buffer (pH = 7.8) buffer. MDC‐incorporated structures were monitored under a Nikon Eclipse E600 epifluorescence microscope (Nikon, Tokyo, Japan), excited by a wavelength of 405 nm and detected at 400–580 nm.

### Electron microscopy

Samples from differently treated leaves were fixed overnight at 4 °C in 3% glutaraldehyde and 0.1 m sodium cacodylate buffer (pH 6.9) and then processed for electron microscopy according to Xu *et al*. (2012a). Ultrathin sections, cut with an ultramicrotome (Ultracut, Reichert Jung), were observed with a transmission electron microscope (TEM 300, Itachi) operating at 75 kV.

### Protein purification and EMSA assay

The tomato ERF5 recombinant protein was prepared according to Zhang *et al*. (2014a,b). Briefly, the full‐length ERF5 CDS PCR product was digested with BamHI and SalI and ligated into the same sites of pET‐32a vector. The recombinant vector was transformed into *E. coli* strain BL21 (DE3). Expression of the recombinant proteins was induced by isopropyl b‐D‐1‐thiogalactopyranoside and purified according to the instructions of the Novagen pET purification system. The probes were shown in Table S1. EMSA of the ERF5‐DNA complexes was performed according to the instructions of electrophoretic mobility shift assay (EMSA) Kit, with SYBR^®^ Green & SYPRO^®^ Ruby EMSA stains (Invitrogen).

### Characterization of promoter activity

To determine the promoter activity, the 1200bp/1700bp fragments of *ATG8d*/*ATG18h* promoter region were amplified using specific primers and fused independently to the β‐d‐glucuronidase (GUS) or luciferase (LUC) reporter gene in the pBI121 vector. Additionally, the cauliflower mosaic virus (CaMV) 35S promoter was fused to GUS or LUC as a control for variation in transformation rate. All constructs were transformed into Agrobacterium strain EHA105. Transient expression was analysed in *N. benthamiana* leaves as described previously (Deng *et al*., 2015). After 12 h of transient transformation, the plants were treated with ACC. Three days after infiltration, GUS and LUC activity was determined as described previously (Deng *et al*., 2015). For drought treatment, the *N. benthamiana* plants were first treated with 0.1 μm ACC for 12 h, then the leaves were transiently transformed with different constructs, after another 12 h, they were treated with 16% PEG6000 for 60 h. GUS and LUC activity was determined as described previously (Deng *et al*., 2015).

### H_2_O_2_ detection and Stomatal conductance

H_2_O_2_ fluorescence and content were conducted as described (Zhu *et al*., 2016a,b).

Stomatal conductance was detected according to Wang *et al*. (2015).

For NADPH oxidase activity analysis, leaf plasma membranes were isolated using a two‐phase aqueous polymer partition system. The NADPH‐dependent superoxide‐generating activity was determined as described previously (Deng *et al*., 2016).

Superoxide and H_2_O_2_ staining were visually detected with nitro blue tetrazolium (NBT) and 3, 3′‐diaminobenzidine (DAB) (Sigma‐Aldrich) according to Zhu *et al*. (2016a,b).

### Enzyme activity assays

SOD CAT and APX activity was measured according to Zhu *et al*. (2016a,b). By monitoring the decrease in absorbance at 290 nm as ascorbate was oxidized.

### Statistical analysis

Data from experiments with three or more mean values were statistically analysed using Duncan multiple range. The difference was considered to be statistically significant at *P* < 0.05.

## Conflict of interests

The authors declare no conflict of interests.

## Supporting information


**Figure S1** Phenotype and stomatal closure in ACC pretreated tomatoes under drought stress.
**Figure S2** ROS accumulation and respiration rate in transgenic lines.
**Figure S3** Lateral buds length in transgenic lines.
**Figure S4** SOD and CAT activity in transgenic tomatoes.
**Figure S5** ACC‐induced autophagic activity and ERF5 transcription under drought stress.
**Figure S6** Silence efficiency of *ATG8d/ATG18h* in WT plants.
**Figure S7** Autophagic activity in *ATG8d/ATG18h*‐silenced plants.
**Figure S8** Silence efficiency of *ATG8d/ATG18h* in *aox19* plants.
**Figure S9** Modified ROS signaling lead to different drought responses in ACC pre‐treated transgenic tomatoes.
**Figure S10** Purified ERF5 protein for EMSA assay.
**Table S1** Primers used in this assay.
