# Supplementary material for: Mitochondrial alternative oxidase‐dependent autophagy involved in ethylene‐mediated drought tolerance in Solanum lycopersicum
Source: Plant Biotechnol J. 2018 May 24;16(12):2063–76. doi: 10.1111/pbi.12939 (PMC6230944; doi:10.1111/pbi.12939)
Supplement: Supplementary file 1 — Figure S1 Phenotype and stomatal closure in ACC pretreated tomatoes under drought stress. Figure S2 ROS accumulation and respiration rate in transgenic lines. Figure S3 Lateral buds length in transgenic lines. Figure S4 SOD and CAT activity in transgenic tomatoes. Figure S5 ACC‐induced autophagic activity and ERF5 transcription under drought stress. Figure S6 Silence efficiency of ATG8d/ATG18h in WT plants. Figure S7 Autophagic activity in ATG8d/ATG18h‐silenced plants. Figure S8 Silence efficiency of ATG8d/ATG18h in aox19 plants. Figure S9 Modified ROS signaling lead to different drought responses in ACC pre‐treated transgenic tomatoes. Figure S10 Purified ERF5 protein for EMSA assay. Table S1 Primers used in this assay. [file PBI-16-2063-s001.pdf]

# Mitochondrial alternative oxidase dependent autophagy involved in ethylene-mediated drought tolerance in *Solanum lycopersicum*

Tong Zhu †1, Lijuan Zou†2, Yan Li †1, Xiuhong Yao[1],Fei Xu[3], Xingguang Deng[1], Dawei Zhang[1]* and Honghui Lin[1]*

[1]Ministry of Education, Key Laboratory for Bio-Resource and Eco-Environment, College of Life Science, Sichuan University, Chengdu, Sichuan, China

[2]Life Science and Technology College and Ecological Security and Protection Key Laboratory of Sichuan Province, Mianyang Normal University, Mianyang 621000, China

[3]Life Science and Biotechnology, Wuhan Bioengineering Institute, Wuhan 430415, China.

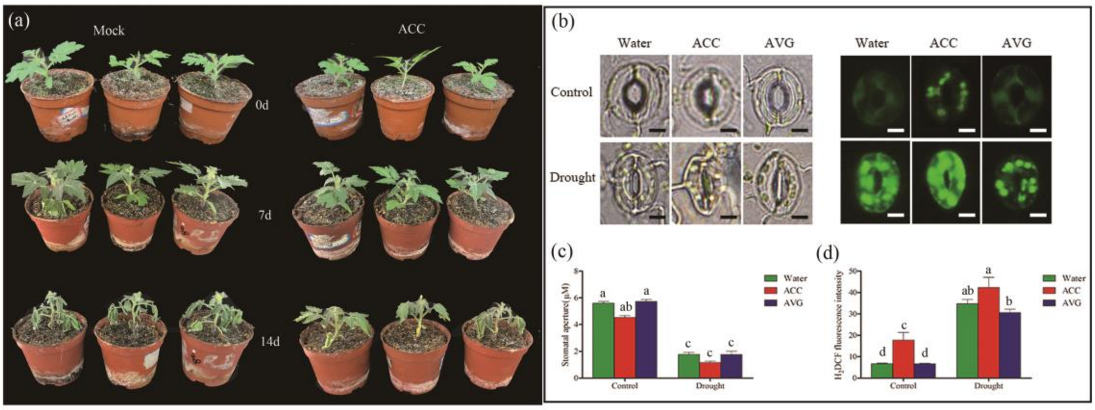


**Figure S1 Phynotype and stomatal closure analysis of tomato seedlings in different pretreatments under drought stress.** (a) Phenotype of tomato plants during drought stress after 0.1 μM ACC pretreatment. The left side was the control group and the right side was the treatment group. Each group has three parallel samples. (b) Stomatal closure in different treatment groups under microscope. (c) Statistics of horizontal distance of stomatal aperture in different treatment groups. (d) Statistics of green fluorescence intensity after $H_2$DCF-DA staining. Bars represent mean and standard deviation of values obtained from three biological repeats. Means with the same letter did not significantly differ at P<0.05 according to Duncan multiple range tests.

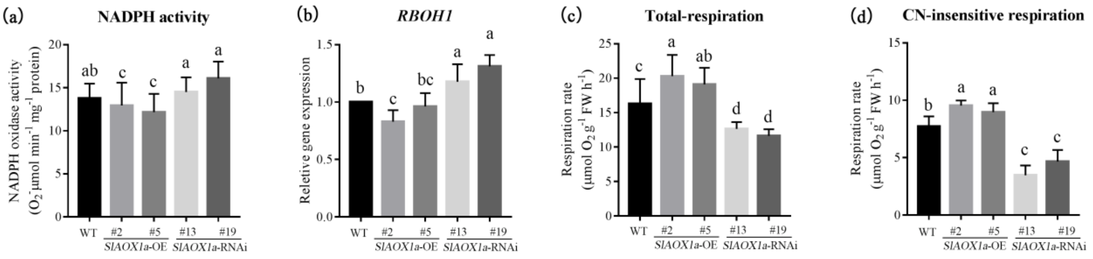


**Figure S2 ROS accumulation and respiration rate in transgenic lines.** NADPH activity (a), *RBOH1*expression (b), total-respiration rate (c) and CN-insensitive respiration rate (d) in transgenic tomatoes. Bars represent mean and standard deviation of values obtained from three biological repeats. Signifcant differences (P<0.05) are denoted by different lowercase letters.

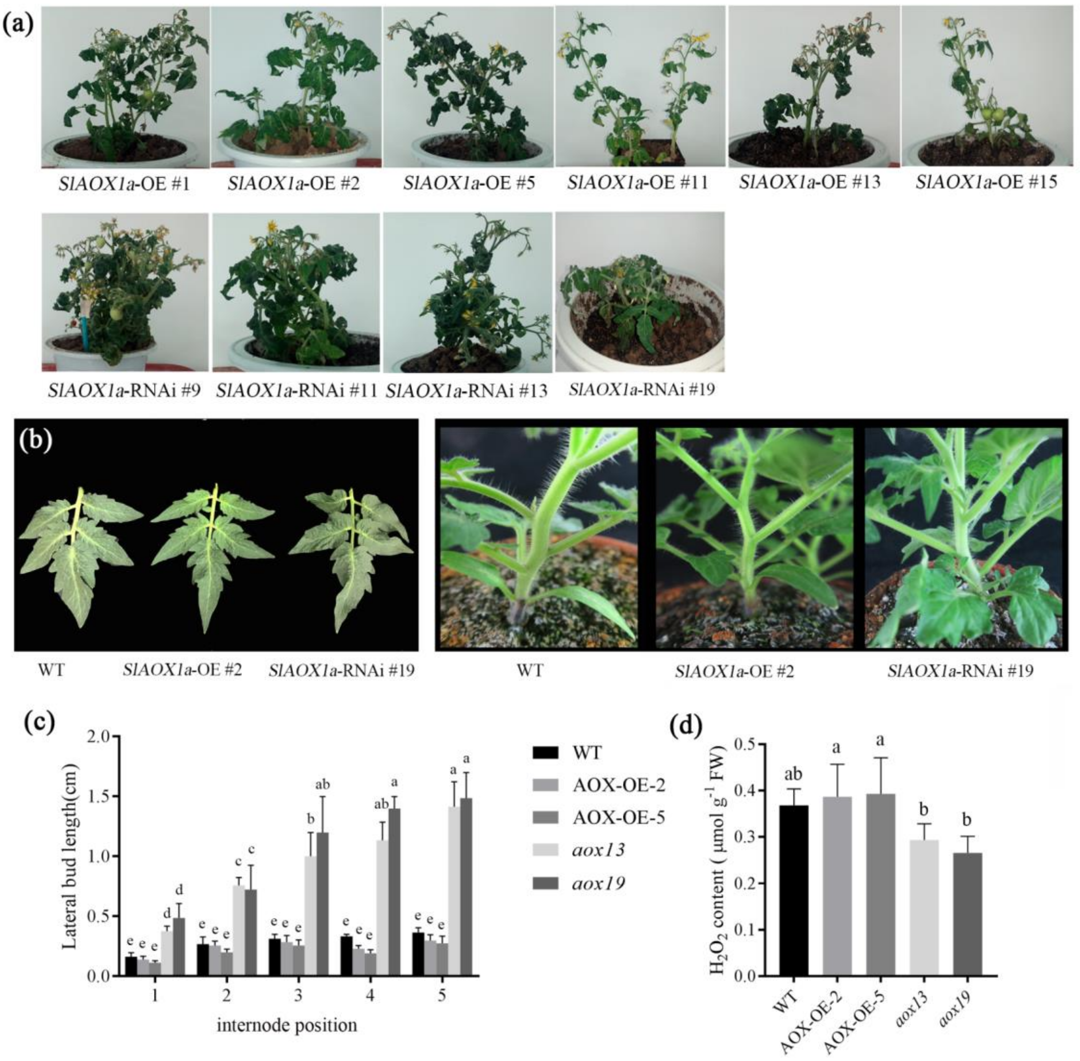

**Figure S3 Developmental changes and lateral buds length in transgenic lines.** (a) Phenotypes of T0 transgenic tomato lines. (b) Leaf color and later bud growth in transgenic tomato plants. Lateral bud length (c) and $H_2O_2$ content (d) were detected in transgenic tomato plants. All data are presented as the means of at least 3 biological replicates (± SE). Means with the same letter did not significantly differ at P<0.05 according to Duncan multiple range tests.

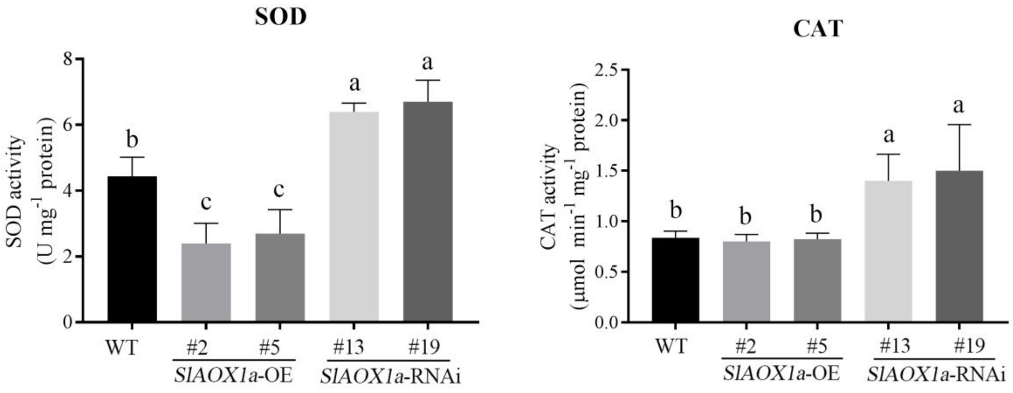


**Figure S4 SOD and CAT activity in transgenic tomatoes.** Bars represent mean and standard deviation of values obtained from three biological repeats. Signifcant differences (P<0.05) are denoted by different lowercase letters.

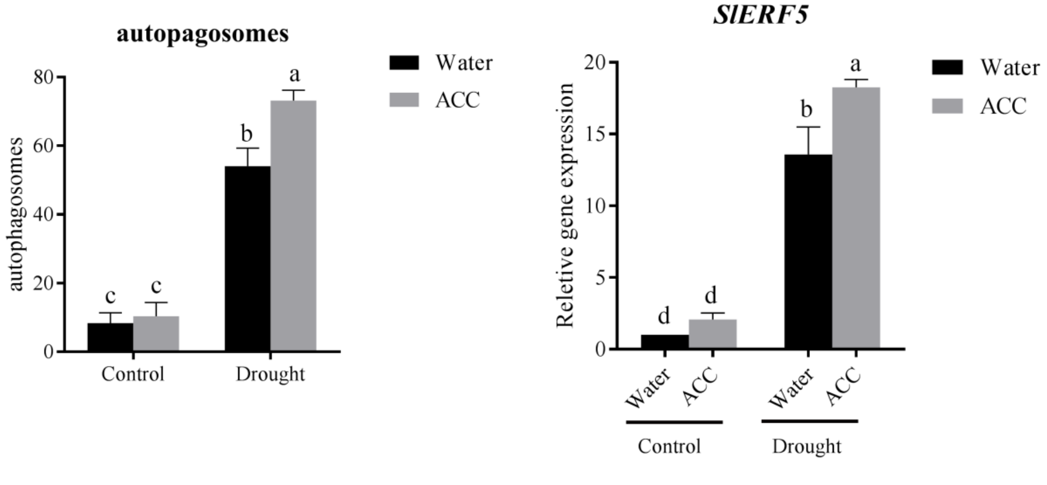


**Figure S5 ACC-induced autophagic activity and *SlERF5* transcription under drought stress.** Bars represent mean and standard deviation of values obtained from three biological repeats. Means with the same letter did not significantly differ at P<0.05 according to Duncan multiple range tests.

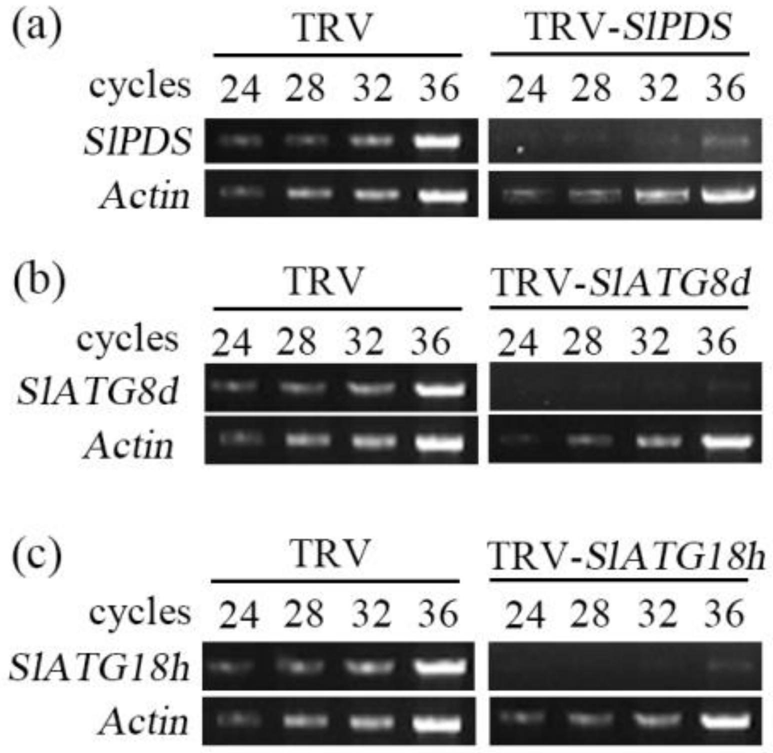


**Figure S6 Silence efficiency of *ATG8d/ATG18h* in WT plants.** (a) Silence efficiency of *SlPDS* in TRV and TRV-*SlPDS* plants. (b) Silence efficiency of *SlATG8d* in TRV and TRV-*SlATG8d* plants. (c) Silence efficiency of *SlATG18h* in TRV and TRV-*SlATG18h* plants. *Actin* was used as control. *Actin* band was the same in TRV plants. The PCR programme cycles was set as 24, 28, 32 and 36, respectively. Three independent experiments were performed with similar results.

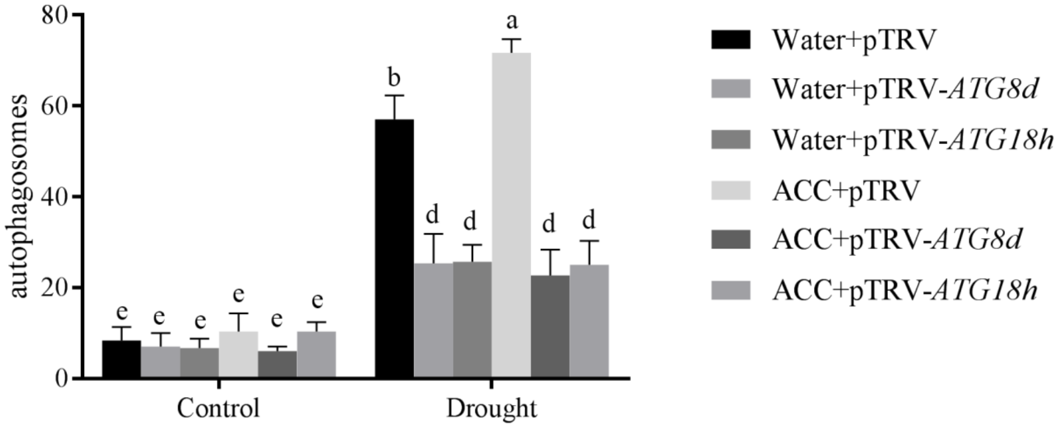


**Figure S7 Autophagic activity in *ATG8d/ATG18h*-silenced plants.** Bars represent mean and standard deviation of values obtained from three biological repeats. Means with the same letter did

not significantly differ at P<0.05 according to Duncan multiple range tests.

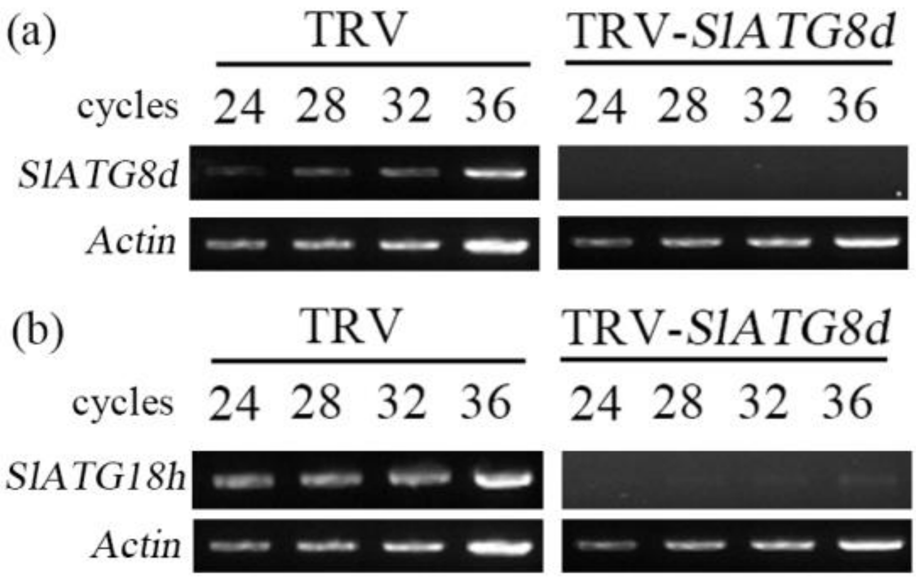


**Figure S8 Silence efficiency of *ATG8d/ATG18h* in *aox19* plants.** (a) Silence efficiency of *SlATG8d* in TRV and TRV-*SlATG8d* plants. (b) Silence efficiency of *SlATG18h* in TRV and TRV-*SlATG18h* plants. The PCR programme cycles was set as 24, 28, 32 and 36 respectively. *Actin* was used as control. *Actin* band was the same in TRV and TRV-*SlATG8d* plants, respectively. Three independent experiments were performed with similar results.

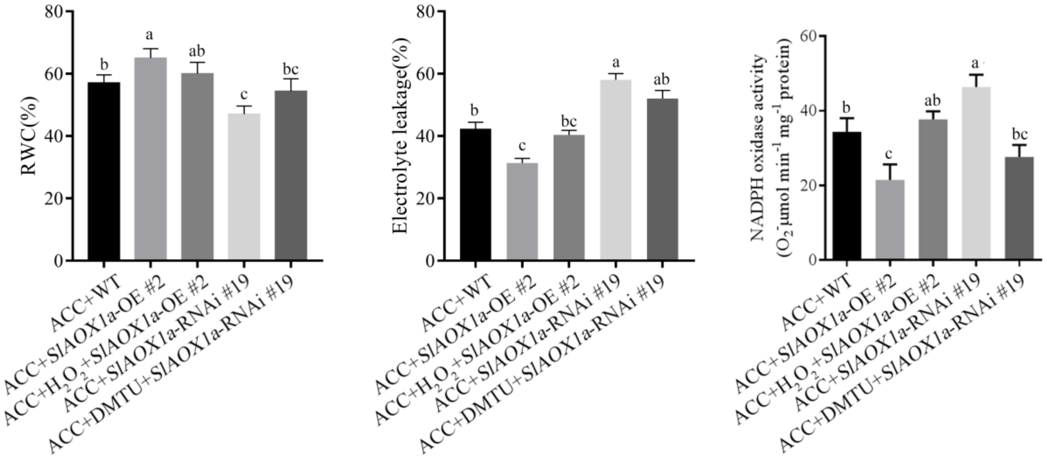


**Figure S9 Modified ROS signaling lead to different drought responses in ACC pre-treated transgenic tomatoes.** Bars represent mean and standard deviation of values obtained from three biological repeats. Signifcant differences (P<0.05) are denoted by different lowercase letters.

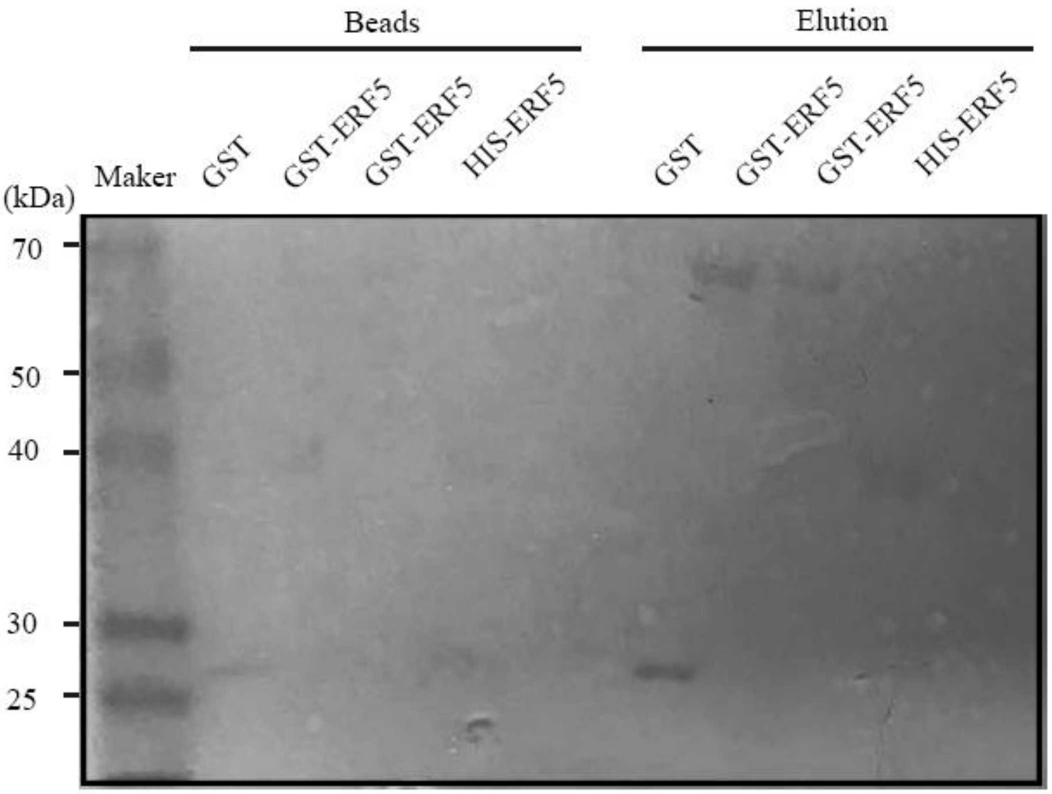


**Figure S10 Purified ERF5 protein for EMSA assay.**

Primers for Transgenic tomato

| Gene | Accession number | Forward primer (5’-3’) | Reverse primer (5’-3’) |
|---|---|---|---|
| *SlAOX1a* | NM_001247188 | ATGATGACCCGTGGAGCA | TGGTGAACCCATAGTGATT |
| *RNAi* | NM_001247188 | AATGGTGGGAGGTATGTT | AGGCAGTCGCCAGTAATC |

Primers for VIGS assay:

| Gene | Accession number | Forward primer (5’-3’) | Reverse primer (5’-3’) |
|---|---|---|---|
| *SlATG8d* | Sl10g006270 | AAGGCTGACAGAAGTGA | GTACGAAACAGACATAAGAT |
| *SlATG18h* | Sl07g064060 | GGAGTGGAGGTAATGGTT | GTTCTTTGCGATTCTGTTT |
| *SlPDS* | NM_001247166 | GCTCTAGACTGGTCACAAACCGATAC | CGGGATCCCACCTCATCTGTCACCCT |

Primers for RT-PCR assay:

| Gene | Accession number | Forward primer (5’-3’) | Reverse primer (5’-3’) |
|---|---|---|---|
| *NPTII:* | | GAGAGGCTATTCGGCTATG | CTCAGAAGAACTCGTCAAGA |
| *35S/NOS* | | CGTAAGGGATGACGCACAA | AAGACCGGCAACAGGATT |

Primers for qRT-PCR

| Gene | Accession number | Forward primer (5’-3’) | Reverse primer (5’-3’) |
|---|---|---|---|
| *SlAOX1a* | AY034148 | GAATGGTGGGAGGTATGT | GTACGAAACAGACATAAGAT |
| *SlAOX1b* | AY034149 | TCCTCCACTGTAAATCCC | GGTAGCCCTTCAACTCAT |
| *SlAOX2* | AY324396 | CGCAAGTTCGAGCACAGT | TAGGCAGTCTCCAGTAGTCAAT |
| *SlATG8d* | Sl10g006270 | AAGGCTGACAGAAGTGA | ACTAACGAACTTGGGTGA |
| *SlATG8f* | Sl08g078820 | GGCAATCATGTCTGCAATCT | TCAAAGCTACAGTTCGCTCAG |
| *SlATG13a* | Sl03g096790 | GATGTCGACACTTCCGATTC | TTGCAGATCCCTGAGAAGAG |
| *SlATG18h* | Sl07g064060 | GGAGTGGAGGTAATGGTT | GTTCTTTGCGATTCTGTTT |
| *SlATG18b* | Sl07g006120 | TTGAGGAGACAGCAACACCT | TGTTCTGATGGTTGACGTTG |
| *SlERF5* | | ATGGGTT CTCCACAAGA | AGAGTCGAACAATACTAT |
| *SlRBOH1* | NM_001330445 | AATAACAAAA CTAACGGATG | GGAGACGTTTGTTTAATAGT |
| *SlATG8d-Promoter* | NM_001247197 | CCGCTCGAGAAATAATCTAGTCACCCTA | CCCAAGCTTAATTCAGTCTTTTCTGTCGATCTCG |
| *SlATG18h-Promoter* | | CCGCTCGAGCTTGTGGCAACTAAATGAAATGAAA | CCCAAGCTTATCACCGTTCTTGATAACCCCTAGG |

Table S1 Primers used in this assay.
